# Assessing the healthcare resource use associated with inappropriate prescribing of inhaled corticosteroids for people with chronic obstructive pulmonary disease (COPD) in GOLD groups A or B: an observational study using the Clinical Practice Research Datalink (CPRD)

**DOI:** 10.1186/s12931-018-0767-2

**Published:** 2018-04-11

**Authors:** James D. Chalmers, Chris Poole, Samantha Webster, Abigail Tebboth, Scott Dickinson, Alicia Gayle

**Affiliations:** 1 Scottish Centre for Respiratory Research, University of Dundee, Ninewells Hospital and Medical School, Dundee, UK; 2 Boehringer Ingelheim Ltd, Bracknell, UK

**Keywords:** Chronic obstructive pulmonary disease, Resource use, Inhaled corticosteroids, Long-acting bronchodilators

## Abstract

**Background:**

Recent recommendations from the Global Initiative for Chronic Obstructive Lung Disease (GOLD) position inhaled corticosteroids (ICS) for use in chronic obstructive pulmonary disease (COPD) patients experiencing exacerbations (≥ 2 or ≥ 1 requiring hospitalisation); i.e. GOLD groups C and D. However, it is known that ICS is frequently prescribed for patients with less severe COPD. Potential drivers of inappropriate ICS use may be historical clinical guidance or a belief among physicians that intervening early with ICS would improve outcomes and reduce resource use. The objective of this study was to compare healthcare resource use in the UK for COPD patients in GOLD groups A and B (0 or 1 exacerbation not resulting in hospitalisation) who have either been prescribed an ICS-containing regimen or a non-ICS-containing regimen.

**Methods:**

Linked data from the Clinical Practice Research Datalink (CPRD) and Hospital Episode Statistics (HES) database were used. For the study period (1 July 2005 to 30 June 2015) a total 4009 patients met the inclusion criteria; 1745 receiving ICS-containing therapy and 2264 receiving non-ICS therapy. Treatment groups were propensity score-matched to account for potential confounders in the decision to prescribe ICS, leaving 1739 patients in both treatment arms. Resource use was assessed in terms of frequency of healthcare practitioner (HCP) interactions and rescue therapy prescribing. Treatment acquisition costs were not assessed.

**Results:**

Results showed no benefit associated with the addition of ICS, with numerically higher all-cause HCP interactions (72,802 versus 69,136; adjusted relative rate: 1.07 [*p* = 0.061]) and rescue therapy prescriptions (24,063 versus 21,163; adjusted relative rate: 1.05 [*p* = 0.212]) for the ICS-containing group compared to the non-ICS group. Rate ratios favoured the non-ICS group for eight of nine outcomes assessed. Outcomes were similar for subgroup analyses surrounding potential influential parameters, including patients with poorer lung function (FEV_1_ <  50% predicted), one prior exacerbation or elevated blood eosinophils.

**Conclusions:**

These data suggest that ICS use in GOLD A and B COPD patients is not associated with a benefit in terms of healthcare resource use compared to non-ICS bronchodilator-based therapy; using ICS according to GOLD recommendations may offer an opportunity for improving patient care and reducing resource use.

**Electronic supplementary material:**

The online version of this article (10.1186/s12931-018-0767-2) contains supplementary material, which is available to authorized users.

## Background

Chronic obstructive pulmonary disease (COPD) remains a significant contributor to healthcare resource use due to its considerable morbidity and mortality. According to data at 2012, an estimated 1.2 million people in the UK had diagnosed COPD, and it is now the third leading cause of death globally [1, 2].

Inhaled bronchodilators, such as long-acting β2 agonists (LABAs) and long-acting muscarinic antagonists (LAMAs), are commonly used for symptom management in patients with COPD [3]. These can be prescribed as monotherapies, in fixed dose combinations or in combination with inhaled corticosteroids (ICS).

LABA+LAMA combinations have been shown to improve lung function and breathlessness, and reduce exacerbations compared to ICS + LABA treatment [4–7]. Further, studies have shown that overuse of ICS therapies is associated with increased risk of complications such as oral candidiasis, hoarse voice, skin bruising, pneumonia and fractures [2, 8–13]. As a result, the Global Initiative for Chronic Obstructive Lung Disease (GOLD) 2017 strategy recommends combination bronchodilator therapy for patients with stable COPD to prevent or reduce symptoms, and that ICS-containing therapies should be reserved for patients categorised as group C or D; i.e. exacerbating patients (≥ 2 or ≥ 1 requiring hospitalisation) [2].

ICS is indicated for the treatment of patients with moderate-to-severe COPD and a history of exacerbations. Despite this, several studies have found that this does not reflect current clinical practice, and ICS is commonly prescribed in patients with less severe COPD [3, 14–17].

Some clinicians have advocated prescribing of ICS to patients with less severe disease in specific subgroups, e.g. those with asthma–COPD overlap features or elevated peripheral blood eosinophil count, with the goal of preventing exacerbations and thereby reducing healthcare resource use. This may be partially attributed to guidance issued by the National Institute of Health and Care Excellence (NICE) in 2010, which recommended ICS + LABA therapy for patients with forced expiratory volume in 1 s (FEV_1_) <  50% predicted and remain breathless or have exacerbations despite maintenance LABA therapy [18]. There is little published evidence on the healthcare resource implications resulting from earlier initiation of ICS. Large randomised controlled trials of ICS use have mainly enrolled patients with severe COPD and frequent exacerbations, and have limited follow-up. Real world data can supplement those from randomised controlled trials by providing more detailed healthcare utilisation and longer-term follow-up.

This study therefore aimed to supplement the existing evidence by investigating the prescribing patterns in the UK for a large cohort of COPD patients in GOLD groups A and B (0 or 1 exacerbation not requiring hospitalisation) [17], and evaluate the impact of ICS use in in terms of healthcare resource utilisation.

## Methods

### Study design, participants, and setting

This was a descriptive, population-based longitudinal study using routinely collected healthcare data provided by the Clinical Practice Research Datalink (CPRD). CPRD contains electronic primary care records from general practice in the UK, and is one of the largest databases of longitudinal medical records from primary care in the world [19]. The quality of data in CPRD is subject to rigorous checks and regular audits, and has been used to conduct a large number of published pharmacoepidemiologic studies [20–22]. Linkage between CPRD and Hospital Episode Statistics (HES) data is also available, enabling individual anonymised patient records to be followed across care sectors.

For inclusion in the study, patients were required to be of acceptable patient standard (as defined by CPRD), registered with “up-to-standard” practices for the period of observation, and eligible for linkage with HES. The study population included patients aged ≥40 years with a new diagnosis of COPD (based on any record of a diagnostic read code for COPD in addition to spirometry confirmation of the diagnosis [FEV_1_/forced vital capacity {FVC} ratio <  0.7]) during the study period (1 July 2005 to 30 June 2015) and classified as GOLD category A/B (captured according to exacerbation history). Exacerbations were defined using a validated CPRD algorithm (antibiotic and oral corticosteroid prescriptions for 5–14 days calculated using date of prescription and drug pack information, or lower respiratory tract infection read code or acute exacerbation read code) [23]. The accuracy of COPD diagnosis in CPRD has been validated [24]. Patients were also required to have a prescription of maintenance bronchodilators within three months of diagnosis, and at least one full year of data prior to the index date (date of the last prescription within three months of diagnosis date). Patients with a diagnostic read code for asthma within one year or those with a record of treatment with a LAMA, LABA, ICS or any combination of these in the year prior to the index date, were excluded. The read codes applied are available in the Additional file 1.

### Outcomes of interest

In order to quantify healthcare resource use in the population under consideration, the following all-cause outcomes were accumulated: number of interactions with a healthcare practitioner (HCP); number of hospital admissions; and number of short-acting bronchodilator prescriptions (i.e. rescue therapy). These endpoints were also assessed according to respiratory-related (as opposed to all-cause) read codes; however, this was considered a supplementary exploratory assessment due to the requirement to considerably restrict read codes to those exclusively associated with COPD (i.e., read codes for breathlessness and chest pain were excluded since these could not be exclusively be attributed to COPD; see Additional file 1). Treatment costs were not considered.

### Analysis

After identification of records meeting inclusion criteria, patients were stratified according to their prescription at index date and followed until the first of: discontinuation of treatment; last record of treatment plus 28 days (to allow for treatment period); date of initiation of an alternative treatment (i.e. switch from ICS- to non-ICS-containing treatment or vice versa) less one day; patient’s transfer out of practice; practice last collection date; end of study period; or death. The ICS group contained patients prescribed ICS as part of maintenance therapy in any combination or alone; the non-ICS group contained patients prescribed any regimen not including ICS.

Patients in the ICS and non-ICS groups were propensity score matched to account for potential biases, as well as to adjust for the difference in individual comorbidities (body mass index [BMI] kg/m^2^, modified Medical Research Council [mMRC] score, FEV_1_ [% predicted], diagnosis of type 2 diabetes, diagnosis of osteoporosis, previous stroke, previous pneumonia, prior exacerbations, Index of Multiple Deprivation [IMD]). Propensity scores were created using a published logistic regression model [17]. Groups were pair matched by propensity score at a ratio of 1:1 using a greedy nearest-neighbour matching algorithm. Pairs were matched on the logit of the propensity score, and calipers of width equal to one standard deviation of the logit of the propensity score were used.

A comparison of baseline characteristics across the two treatment groups was performed using Χ^2^ or T-tests and paired T-tests and McNemar tests for matched comparisons, as appropriate. Prevalence was calculated for binary or categorical characteristics, and mean and standard deviation for continuous characteristics. Missing data were quantified for each variable. For the majority of variables, data were considered to be missing at random (unlikely to bias the estimated results) and classified as unknown.

Outcomes of interest were compared for the ICS and non-ICS groups, with subgroup analyses assessing the impact of potentially relevant confounders; this included group A/B patients with FEV_1_ <  50% predicted, one prior exacerbation (i.e. removal of patients that had not experienced an exacerbation) and an eosinophil count > 0.3 × 10^9^/L (selected based on recent literature that suggested a benefit of ICS in patients with an eosinophil count above this level) [17, 25, 26]. Rate ratios were estimated using zero-inflated negative binomial regression of the number of events, in order to take into account individuals more prone to repeated events and provide more conservative confidence intervals and *p* values. We compared a univariate analysis with the fully adjusted model.

## Results

**Fig. 1:**
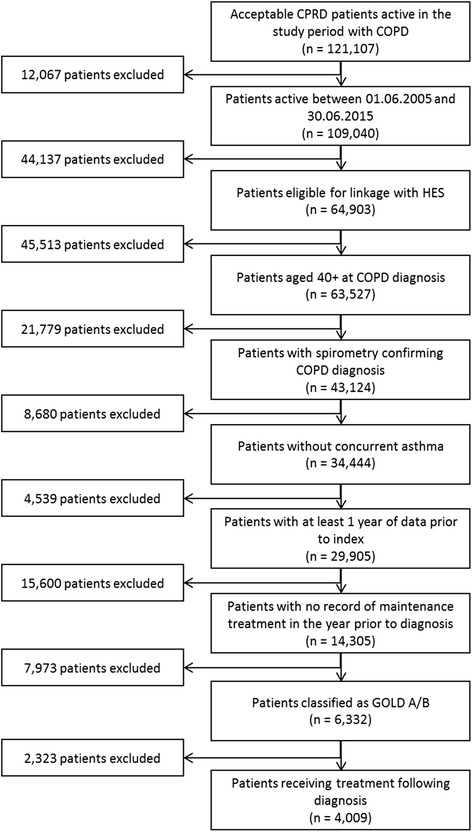
Patient selection flow chart. COPD, chronic obstructive pulmonary disease; CPRD, Clinical Practice Research Datalink; GOLD, Global Initiative for Chronic Obstructive Lung Disease; HES, Hospital Episode Statistics; ICS, inhaled corticosteroid

A total of 4009 patients with newly diagnosed GOLD A/B COPD and a prescription for maintenance bronchodilation treatment within three months of diagnosis were identified (see Fig. 1).

**Fig. 2:**
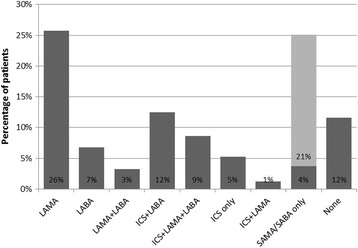
Treatments prescribed within three months of COPD diagnosis. COPD, chronic obstructive pulmonary disease; ICS, inhaled corticosteroid; LABA, long-acting β2 agonist; LAMA, long-acting muscarinic antagonist; SABA, short-acting β_2_ agonist; SAMA, short-acting muscarinic antagonist

The treatments prescribed to patients within three months of COPD diagnosis are displayed in Fig. 2; 44% of the cohort was prescribed an ICS-containing regimen.

There was no statistically significant difference between most groups prior to propensity score matching; following matching, any differences between groups were controlled for. After matching, 531 patients were excluded, leaving a total matched-cohort size of 3478.

On average, patients had 31 months of follow-up (standard deviation [SD] 24 months). Total patient years of follow up were 4348 in the ICS group versus 4686 in the non-ICS group. As the mean difference in follow-up time was minimal, no adjustment was made in the subsequent analysis.

Baseline characteristics of the study population pre- and post-matching are shown in Table 1. In the total matched cohort, there were more male than female patients (60.5% versus 39.5%) and the mean age was 67.8 years (SD: 10 years). On average, patients were diagnosed between 2009 and 2013 (mean: 2011; SD: 2 years). Just under half (47.9%) had a BMI greater than 25 kg/m^2^, and 42.4% were current smokers. Similar proportions of patients had comorbid type 2 diabetes (15.4%), asthma diagnosed more than one year prior to COPD diagnosis (16.6%) and an eosinophil count > 0.3 × 10^9^/L (18.2%).

**Table 1:** Patient baseline demographics and characteristics pre- and post-matching

Parameter	Pre-matching			Post matching		
	ICS (*N* = 1745)	Non-ICS (*N* = 2264)	*P* value	ICS (*N* = 1739)	Non-ICS (*N* = 1739)	*P* value
Female, *n* (%)	679 (38.9)	906 (40.0)	0.494	676 (38.9)	697 (40.1)	0.480
Mean age, years (SD)	67.6 (10.3)	68.0 (10.3)	0.237	67.6 (10.3)	67.9 (10.2)	0.480
Mean BMI, kg/m^2^ (SD)	27 (5.8)	27 (5.8)	0.4189	27 (5.8)	27 (6.0)	0.3166
Mean FEV_1_ predicted, % (SD)	57 (19.4)	61 (18.5)	< 0.001	57 (19.4)	61 (18.5)	< 0.001
Age category, n (%)						
40–44 years	29 (1.7)	26 (1.1)	0.321	27 (1.6)	21 (1.2)	0.143
45–54 years	171 (9.8)	201 (8.9)		171 (9.8)	153 (8.8)	
55–64 years	455 (26.1)	618 (27.3)		454 (26.1)	479 (27.5)	
65–74 years	628 (36.0)	777 (34.3)		626 (36.0)	603 (34.7)	
75–80 years	275 (15.8)	369 (16.3)		274 (15.8)	270 (15.5)	
> 80 years	187 (10.7)	273 (12.1)		187 (10.8)	213 (12.2)	
BMI category, n (%)						
Underweight (< 18.5)	79 (4.5)	78 (3.4)	0.076	79 (4.5)	55 (3.2)	0.235
Normal (18.5–24.9)	512 (29.3)	662 (29.2)		512 (29.4)	509 (29.3)	
Overweight (25–29.9)	436 (25.0)	629 (27.8)		434 (25.0)	484 (27.8)	
Obese (30–39.9)	361 (20.7)	418 (18.5)		358 (20.6)	325 (18.7)	
Severely obese (≥ 40)	26 (1.5)	47 (2.1)		26 (1.5)	39 (2.2)	
Missing	331 (19.0)	430 (19.0)		330 (19.0)	327 (18.8)	
Smoking status, *n* (%)						
Current	726 (41.6)	994 (43.9)	0.097	725 (41.7)	750 (43.1)	0.513
Non-smoker/never smoked	90 (5.2)	85 (3.8)		88 (5.1)	67 (3.9)	
Ex-smoker	773 (44.3)	972 (42.9)		770 (44.3)	761 (43.8)	
Missing	156 (8.9)	213 (9.4)		156 (9.0)	161 (9.3)	
IMD quintile, *n* (%)						
I	292 (16.7)	322 (14.2)	0.175	291 (16.7)	249 (14.3)	0.254
II	326 (18.7)	411 (18.2)		325 (18.7)	313 (18.0)	
III	336 (19.3)	438 (19.3)		334 (19.2)	352 (20.2)	
IV	390 (22.3)	526 (23.2)		388 (22.3)	387 (22.3)	
V	399 (22.9)	565 (23.2)		399 (22.9)	436 (25.1)	
2017 GOLD classification, *n* (%)						
GOLD A	1044 (59.8)	1479 (65.3)	< 0.001	1044 (60.0)	1068 (61.4)	0.356
GOLD B	701 (40.2)	785 (34.7)		695 (40.0)	671 (38.6)	
Comorbidities, *n* (%)						
Eosinophilia (> 0.4 × 10^9^/L)	119 (6.8)	153 (6.8)	0.933	119 (6.8)	127 (7.3)	0.942
Eosinophilia (> 0.3 × 10^9^/L)	326 (18.7)	399 (17.6)	0.683	326 (18.7)	307 (17.7)	0.822
Eosinophilia (read code)	< 5 (−)	< 5 (−)	0.214	< 5 (−)	< 5 (−)	0.942
Asthma diagnosed > 1 year prior	310 (17.8)	304 (13.4)	< 0.001	304 (17.5)	273 (15.7)	0.093
Type II diabetes	271 (15.5)	339 (15.0)	0.627	270 (15.5)	267 (15.4)	0.888
Osteoporosis/osteopenia	227 (13.0)	286 (12.6)	0.724	227 (13.1)	225 (12.9)	0.921
Stroke	118 (6.8)	144 (6.4)	0.610	117 (6.7)	115 (6.6)	0.893
Myocardial infarction	166 (9.5)	238 (10.5)	0.297	166 (9.5)	166 (9.5)	1.000
Pneumonia	357 (20.5)	443 (19.6)	0.484	356 (20.5)	356 (20.5)	1.000
Prior exacerbations	711 (40.7)	895 (39.5)	0.437	709 (40.8)	705 (40.5)	0.890

*BMI* body–mass index, *FEV*_*1*_ forced expiratory volume in 1 s, *GOLD* Global Initiative for Chronic Obstructive Lung Disease, *ICS* inhaled corticosteroid, *IMD* Index of Multiple Deprivation - where I is most deprived, *SD* standard deviation

Table 2 shows the total events in the ICS and non-ICS groups. Fewer short-acting therapy prescriptions were administered in the non-ICS group (*p* <  0.05). All outcomes, with the exception of respiratory-specific hospital interactions (with a difference of 64 events), were numerically higher for the ICS-containing group. Although not statistically significant, ‘all HCP interactions’ approached significance, likely driven by the difference in GP visits between the groups.

**Table 2:** Healthcare resource use during study follow-up

Outcome	Total		ICS versus non-ICS			
			Adjusted		Unadjusted	
	ICS	Non-ICS	Relative rate (95% CI)	*P* value*	Relative rate (95% CI)	*P* value*
Short-acting therapy prescriptions	24,063	21,163	1.05 (0.97–1.14)	0.212	1.09 (1.01–1.18)	0.026
All-cause interactions						
All HCP	72,802	69,136	1.07 (1.00–1.14)	0.061	1.05 (0.98–1.13)	0.156
GP	55,927	52,947	1.07 (1.00–1.14)	0.063	1.06 (0.98–1.13)	0.129
Outpatient	13,497	12,989	1.01 (0.94–1.08)	0.802	1.04 (0.93–1.16)	0.485
Hospital	3378	3200	1.05 (0.93–1.19)	0.434	1.06 (0.94–1.19)	0.377
Respiratory-related interactions						
All HCP^a^	20,555	19,543	1.04 (0.98–1.12)	0.209	1.05 (0.98–1.13)	0.154
Routine GP	16,391	15,409	1.05 (0.99–1.13)	0.127	1.06 (0.99–1.14)	0.078
Unplanned GP	2166	2085	1.04 (0.92–1.18)	0.506	1.04 (0.92–1.17)	0.530
Outpatient	247	234	1.04 (0.78–1.39)	0.809	1.06 (0.80–1.40)	0.705
Hospital	1751	1815	0.95 (0.83–1.09)	0.465	0.96 (0.84–1.10)	0.603

*Significant at α = 0.05; ^a^GP + outpatient + hospital

*CI* confidence interval, *GP* general practitioner, *HCP* healthcare practitioner, *ICS* inhaled corticosteroid

**Fig. 3:**
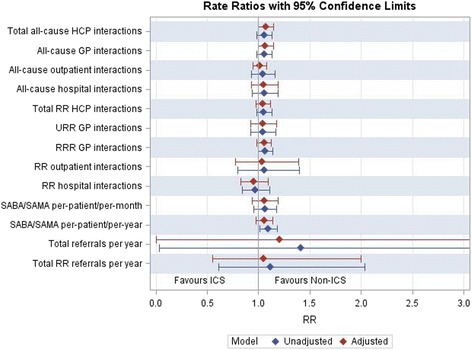
Forest plot of rate ratios for outcomes assessed. HCP, healthcare practitioner; GP, General Practitioner; RR, respiratory-related; RRR, routine respiratory-related; SABA, short-acting β_2_ agonist; SAMA, short-acting muscarinic agonist; URR, unplanned respiratory-related

The direction of relative rates favoured non-ICS containing therapy in all outcomes, with the exception of respiratory-specific hospital interactions (Fig. 3).

**Fig. 4:**
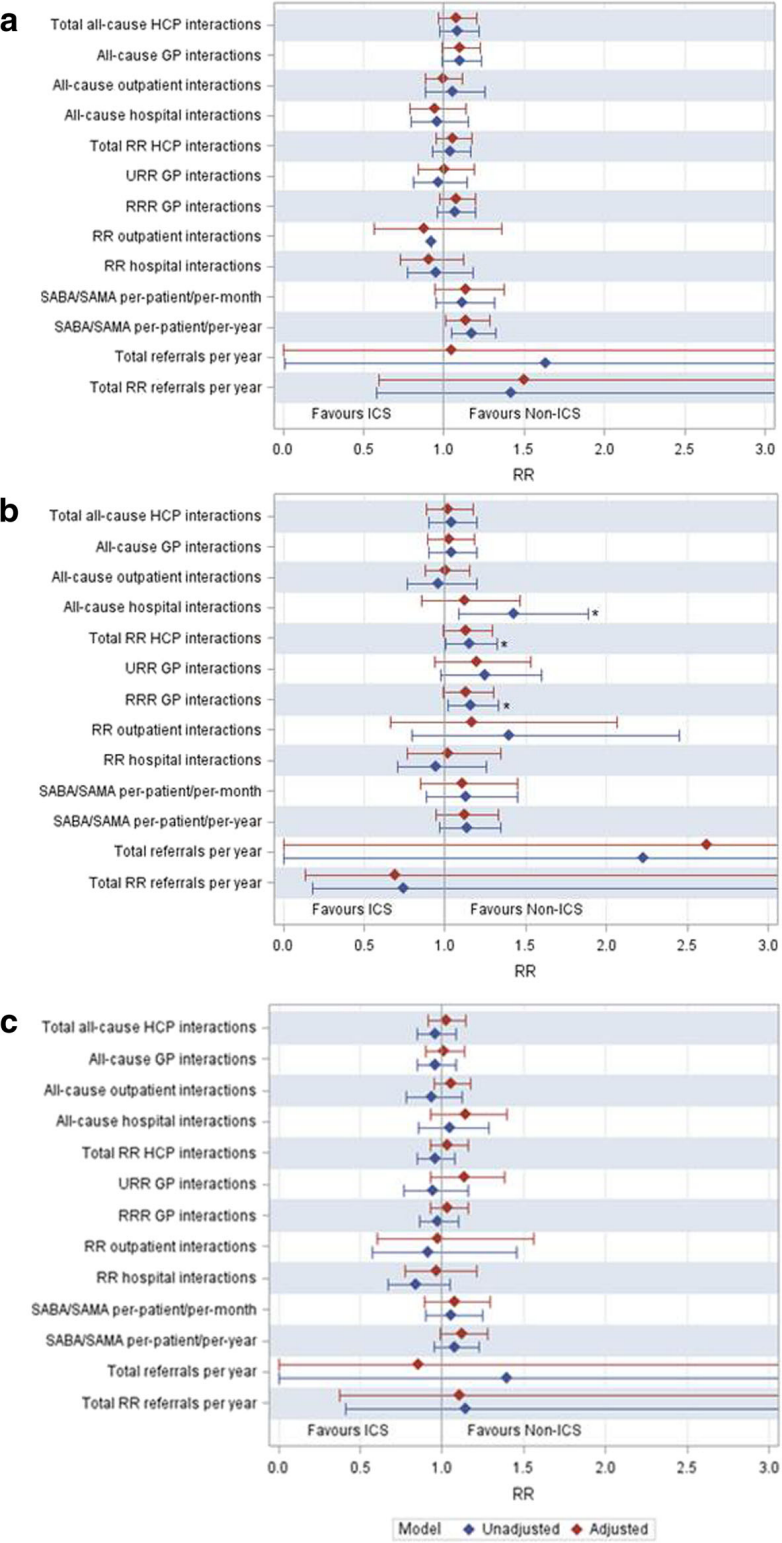
Rate ratios for subgroup analyses. **a** Patients with one previous exacerbation. **b** Patients with an eosinophil level >  0.3 × 109/L. **c** Patients with FEV1 <  50% predicted. HCP, healthcare practitioner; GP, General Practitioner; RR, respiratory-related; RRR, routine respiratory-related; SABA, short-acting β2 agonist; SAMA, short-acting muscarinic agonist; URR, unplanned respiratory-related

Subgroup analyses showed similar trends, with adjusted outcome relative rates generally favouring the non-ICS group. Figure 4 demonstrates this for three key patient subgroups.

## Discussion

CPRD, when linked to HES, provides an important repository of data regarding the diagnosis, treatment and ongoing care of UK patients from the primary to the secondary care setting. According to analysis of this data, almost half of GOLD A/B COPD patients were prescribed an ICS-containing regimen between 2005 and 2015. Despite the widespread use of ICS, no significant differences in rate of healthcare resource use were identified in this study.

In pertinent subgroups of GOLD A/B patients in which escalation to ICS is sometimes advocated in clinical practice, i.e. those who have experienced an exacerbation, have poorer lung function or have elevated eosinophils, the benefit is uncertain. Recent trials of ICS have enrolled patients with a history of only one exacerbation in the prior year (which results in a classification of GOLD A/B). Recent evidence, including from the FLAME study, has demonstrated that bronchodilators can be equally or more effective in preventing exacerbations compared to ICS/LABA [5]. Blood eosinophil counts have been advocated as a biomarker to predict ICS response, and influential authors have advocated ICS use in patients with eosinophil counts > 2% or >  0.3 × 10^9^/L. There are caveats regarding this analysis, specifically that most evidence for prediction of ICS response from eosinophil counts is from post-hoc analyses of prior studies, and most studies enrolled severe patients with a history of frequent exacerbations. For patients with poorer lung function, there is a conflict between the 2010 NICE guidelines [18], which advocate ICS/LABA use for patients with FEV_1_ <  50% predicted, and the GOLD 2017 strategy [2], which promotes ICS use only for patients with two or more exacerbations (or one hospital admission) in the previous year.

It should be noted that this study was not designed to observe the clinical impact of ICS and its potential benefit to some patients; however, the finding that these key patient subgroups do not seem to experience any benefit from initial ICS therapy in terms of HCP interactions is important in view of the current debate on the role of ICS. The rationale for the addition of ICS in these patients, which is contrary to GOLD guidelines [2], should be considered in the context of these findings.

Limitations in read coding meant that conclusions were more difficult to draw for respiratory-related outcomes, and results of this analysis should be interpreted with caution; however, results appear supportive of the primary findings.

On visual inspection, baseline demographics and clinical characteristics of the treatment groups are considered representative of those seen in clinical practice. In terms of patient numbers, a considerable proportion of the starting cohort were excluded at the point of requiring a read code for confirmed spirometry; a record of spirometry confirmed COPD is considered essential to ensure the analysis cohort represented true COPD patients (and not those that actually had asthma or other respiratory condition) [24]. In spite of this, patient numbers were large enough to statistically power the analysis.

Prior to analysis, the matching process attempted to correct for potential biases; however, common to all CPRD analyses, certain biases (e.g. practitioner read code fluency) cannot be ruled out. It is, however, considered that the results of this study are largely generalisable to the UK.

## Conclusions

Despite marketing authorisation, clinical rationale and guideline support for reserving ICS use in more severe patients, ICS is commonly prescribed in patients without exacerbating COPD [2, 3, 8–13, 15–17]. This study demonstrates that there is no reduction in healthcare resource use, rather results tended toward an increase, when ICS is prescribed on top of maintenance bronchodilation. This held for important patient subgroups, e.g. those with FEV_1_ <  50% predicted, a history of exacerbations and an eosinophil count > 0.3 × 10^9^/L. Results suggest that, within the UK healthcare system, there may be opportunity to improve patient care and potentially reduce resource use for patients receiving maintenance treatment for GOLD A/B COPD.

## Additional file

Additional file 1: Codes used to identify patients, exposure and outcomes. (DOCX 122 kb)

## Abbreviations

BMI: Body–mass index
CI: Confidence interval
COPD: Chronic obstructive pulmonary disease
CPRD: Clinical Practice Research Datalink
FEV_1_: Forced expiratory volume in 1 s
FVC: Forced vital capacity
GOLD: Global Initiative for Chronic Obstructive Lung Disease
GP: General Practitioner
HCP: Healthcare practitioner
HES: Hospital Episode Statistics
ICS: Inhaled corticosteroid
IMD: Index of multiple deprivation
LABA: Long-acting β2 agonist
LAMA: Long-acting muscarinic antagonist
mMRC: Modified Medical Research Council
NICE: National Institute of Health and Care Excellence
RR: Respiratory-related
RRR: Routine respiratory-related
SABA: Short-acting β_2_ agonist
SAMA: Short-acting muscarinic antagonist
SD: Standard deviation
URR: Unplanned respiratory-related

## References

[1] Snell N, Strachan D, Hubbard R, Gibson J, Gruffydd-Jones K, Jarrold I (2016). S32 Epidemiology of chronic obstructive pulmonary disease (COPD) in the uk: findings from the british lung foundation’s ‘respiratory health of the nation’ project. Thorax.

[2] Vogelmeier CF, Criner GJ, Martinez FJ, Anzueto A, Barnes PJ, Bourbeau J (2017). Global strategy for the diagnosis, management, and prevention of chronic obstructive lung disease 2017 report. GOLD executive summary. Am J Respir Crit Care Med.

[3] Jones R, Freemantle N, Miravitlles M, Bruselle G, Gruffydd-Jones K, Baldwin M, et al. Inappropriate prescriptions following initial COPD diagnosis. Eur Respir J. 2013;42(Suppl 57):2391.

[4] Vogelmeier CF, Bateman ED, Pallante J, Alagappan VK, D'Andrea P, Chen H, et al. Efficacy and safety of once-daily QVA149 compared with twice-daily salmeterol-fluticasone in patients with chronic obstructive pulmonary disease (ILLUMINATE): a randomised, double-blind, parallel group study. Lancet Respir Med. 2013;(1, 1):51–60. 10.1016/S2213-2600(12)70052-8 24321804

[5] Wedzicha JA, Banerji D, Chapman KR, Vestbo J, Roche N, Ayers RT (2016). Indacaterol–Glycopyrronium versus salmeterol–fluticasone for COPD. N Engl J Med.

[6] Magnussen H, Disse B, Rodriguez-Roisin R, Kirsten A, Watz H, Tetzlaff K (2014). Withdrawal of inhaled glucocorticoids and exacerbations of COPD. N Engl J Med.

[7] Beeh KM, Derom E, Echave-Sustaeta J, Gronke L, Hamilton A, Zhai D (2016). The lung function profile of once-daily tiotropium and olodaterol via Respimat((R)) is superior to that of twice-daily salmeterol and fluticasone propionate via Accuhaler((R)) (ENERGITO((R)) study). Int J Chron Obstruct Pulmon Dis.

[8] Calverley PMA, Anderson JA, Celli B, Ferguson GT, Jenkins C, Jones PW (2007). Salmeterol and fluticasone propionate and survival in chronic obstructive pulmonary disease. N Engl J Med.

[9] Dransfield MT, Bourbeau J, Jones PW, Hanania NA, Mahler DA, Vestbo J (2013). Once-daily inhaled fluticasone furoate and vilanterol versus vilanterol only for prevention of exacerbations of COPD: two replicate double-blind, parallel-group, randomised controlled trials. Lancet Respir Med.

[10] Loke YK, Cavallazzi R, Singh S (2011). Risk of fractures with inhaled corticosteroids in COPD: systematic review and meta-analysis of randomised controlled trials and observational studies. Thorax.

[11] Kew KM, Seniukovich A (2014). Inhaled steroids and risk of pneumonia for chronic obstructive pulmonary disease. The Cochrane database of systematic reviews.

[12] Crim C, Dransfield MT, Bourbeau J, Jones PW, Hanania NA, Mahler DA (2015). Pneumonia risk with inhaled fluticasone furoate and vilanterol compared with vilanterol alone in patients with COPD. Annals of the American Thoracic Society.

[13] Yang IA, Fong KM, Sim EH, Black PN, Lasserson TJ (2007). Inhaled corticosteroids for stable chronic obstructive pulmonary disease. The Cochrane database of systematic reviews.

[14] Tariq SM, Thomas EC (2017). Maintenance therapy in COPD: time to phase out ICS and switch to the new LAMA/LABA inhalers?. Int J Chron Obstruct Pulmon Dis.

[15] Seaman J, Leonard AC, Panos RJ (2010). Health care utilization history, GOLD guidelines, and respiratory medication prescriptions in patients with COPD. Int J Chron Obstruct Pulmon Dis.

[16] Gruffydd-Jones K, Brusselle G, Jones R, Miravitlles M, Baldwin M, Stewart R (2016). Changes in initial COPD treatment choice over time and factors influencing prescribing decisions in UK primary care: in UK primary care: a real-world, retrospective, observational. Npj Primary Care Respiratory Medicine.

[17] Chalmers JD, Tebboth A, Gayle A, Ternouth A, Ramscar N. Determinants of initial inhaled corticosteroid use in patients with GOLD A/B COPD: a retrospective study of UK general practice. npj Primary Care Respiratory Medicine. 2017; Article in press 10.1038/s41533-017-0040-z PMC5491501 28663549

[18] National Institute for Health and Care Excellence. Chronic obstructive pulmonary disease in over 16s: diagnosis and management, Clinical guideline [CG101]. 2010. https://www.nice.org.uk/guidance/cg101. Accessed 12 December 2017. 31211541

[19] Kousoulis AA, Rafi I, de Lusignan S (2015). The CPRD and the RCGP: building on research success by enhancing benefits for patients and practices. Br J Gen Pract.

[20] Herrett E, Thomas SL, Schoonen WM, Smeeth L, Hall AJ (2010). Validation and validity of diagnoses in the general practice research database: a systematic review. Br J Clin Pharmacol.

[21] Khan NF, Harrison SE, Rose PW (2010). Validity of diagnostic coding within the general practice research database: a systematic review. Br J Gen Pract.

[22] Jick SS, Kaye JA, Vasilakis-Scaramozza C, Garcia Rodriguez LA, Ruigomez A, Meier CR (2003). Validity of the general practice research database. Pharmacotherapy.

[23] Rothnie KJ, Müllerová H, Hurst JR, Smeeth L, Davis K, Thomas SL (2016). Validation of the recording of acute exacerbations of COPD in UK primary care electronic healthcare records. PLoS One.

[24] Quint JK, Müllerova H, DiSantostefano RL, Forbes H, Eaton S, Hurst JR, et al. Validation of chronic obstructive pulmonary disease recording in the clinical practice research datalink (CPRD-GOLD). BMJ Open. 2014;4(7) 10.1136/bmjopen-2014-005540 PMC4120321 25056980

[25] Watz H, Tetzlaff K, Wouters EF, Kirsten A, Magnussen H, Rodriguez-Roisin R (2016). Blood eosinophil count and exacerbations in severe chronic obstructive pulmonary disease after withdrawal of inhaled corticosteroids: a post-hoc analysis of the WISDOM trial. Lancet Respir Med.

[26] Brusselle GG, Bracke K, Lahousse L (2015). Targeted therapy with inhaled corticosteroids in COPD according to blood eosinophil counts. Lancet Respir Med.

