# Supplementary material for: Assessing the healthcare resource use associated with inappropriate prescribing of inhaled corticosteroids for people with chronic obstructive pulmonary disease (COPD) in GOLD groups A or B: an observational study using the Clinical Practice Research Datalink (CPRD)
Source: Respir Res. 2018 Apr 11;19:63. doi: 10.1186/s12931-018-0767-2 (PMC5896104; doi:10.1186/s12931-018-0767-2)
Supplement: Supplementary file 1 — Codes used to identify patients, exposure and outcomes. (DOCX 122 kb) [file 12931_2018_767_MOESM1_ESM.docx]

Supplementary material: Codes used to identify patients, exposure and outcomes

Contents

[Conditions 2](#_Toc507080841)

[Chronic obstructive pulmonary disease (COPD) 2](#_Toc507080842)

[Exacerbation 3](#_Toc507080843)

[Lower respiratory tract infection 3](#_Toc507080844)

[Cough 5](#_Toc507080845)

[Breathlessness 5](#_Toc507080846)

[Sputum 6](#_Toc507080847)

[Asthma 7](#_Toc507080848)

[Treatments 10](#_Toc507080849)

[LABA 10](#_Toc507080850)

[LAMA 11](#_Toc507080851)

[Inhaled corticosteroid 11](#_Toc507080852)

[Consultations 17](#_Toc507080853)

# Conditions

## Chronic obstructive pulmonary disease (COPD)

| **Medical code** | **Read term** |
| --- | --- |
| 794 | Emphysema |
| 998 | Chronic obstructive airways disease |
| 1001 | Chronic obstructive pulmonary disease |
| 3243 | Chronic bronchitis |
| 5710 | Chronic obstructive airways disease NOS |
| 9876 | Severe chronic obstructive pulmonary disease |
| 10802 | Moderate chronic obstructive pulmonary disease |
| 10863 | Mild chronic obstructive pulmonary disease |
| 11287 | Chronic obstructive pulmonary disease annual review |
| 12166 | Other specified chronic obstructive airways disease |
| 14798 | Emphysematous bronchitis |
| 15157 | Chronic bronchitis NOS |
| 15782 | Chronic pulmonary heart disease NOS |
| 16410 | Other emphysema NOS |
| 21061 | Chronic obstruct pulmonary dis with acute lower resp infectn |
| 23492 | Chronic bullous emphysema NOS |
| 25603 | Simple chronic bronchitis |
| 26082 | Chronic pulmonary oedema |
| 26306 | Chronic bullous emphysema |
| 27819 | Obstructive chronic bronchitis |
| 33450 | Emphysema NOS |
| 37247 | Chronic obstructive pulmonary disease NOS |
| 40788 | Other emphysema |
| 44525 | Obstructive chronic bronchitis NOS |
| 45770 | Chronic obstructive pulmonary disease disturbs sleep |
| 45771 | Chronic obstructive pulmonary disease does not disturb sleep |
| 46578 | Panlobular emphysema |
| 54893 | Compensatory emphysema |
| 56860 | Segmental bullous emphysema |
| 60188 | Giant bullous emphysema |
| 61118 | Simple chronic bronchitis NOS |
| 64721 | Chronic emphysema due to chemical fumes |
| 65733 | [X]Other specified chronic obstructive pulmonary disease |
| 66043 | Other chronic bronchitis |
| 66058 | [X]Other emphysema |
| 67040 | Other specified chronic obstructive pulmonary disease |
| 68066 | Other chronic bronchitis NOS |
| 68662 | Zonal bullous emphysema |
| 70787 | Atrophic (senile) emphysema |
| 93568 | Very severe chronic obstructive pulmonary disease |
| 99536 | Bullous emphysema with collapse |

| **ICD-10** | **Term** |
| --- | --- |
| J44 | Chronic Obstructive Pulmonary Disease |
| J44.0 | Chronic obstructive pulmonary disease with acute lower respiratory infection |
| J44.1 | Chronic obstructive pulmonary disease with acute exacerbation, unspecified |
| J44.8 | Other specified chronic obstructive pulmonary diseas |
| J44.9 | Chronic obstructive pulmonary disease, unspecified |
| J43 | Emphysema |
| J42 | Unspecified chronic bronchitis |

## Exacerbation

Exacerbations were defined using a validated algorithm for acute exacerbations in CPRD data:

- antibiotic and oral corticosteroid (OCS) prescriptions for 5–14 days calculated using date of prescription and drug pack information
- or lower respiratory tract infection (LRTI) READ code
- or acute exacerbation (AECOPD) READ code

| **Medical code** | **Read term** |
| --- | --- |
| 19106 | COPD accident and emergency |
| 11019 | admit COPD emergency |
| 1446 | acute exacerbation of COPD |
| 7884 | COPD with acute exacerbation |
| 101042 | Issue of chronic obstructive pulmonary disease rescue pack |

## Lower respiratory tract infection

| **Medical code** | **Read term** |
| --- | --- |
| 68 | Chest infection |
| 312 | Acute bronchitis |
| 556 | Influenza |
| 1019 | Acute bronchiolitis |
| 1382 | Acute viral bronchitis unspecified |
| 2157 | Flu like illness |
| 2476 | Chest cold |
| 2581 | Chest infection NOS |
| 3358 | Lower resp tract infection |
| 5947 | Influenza like illness |
| 5978 | Acute wheezy bronchitis |
| 6124 | Acute lower respiratory tract infection |
| 6181 | Obliterating fibrous bronchiolitis |
| 8980 | Influenza-like symptoms |
| 9043 | Acute pneumococcal bronchitis |
| 11072 | Acute purulent bronchitis |
| 14791 | Influenza with gastrointestinal tract involvement |
| 15774 | Influenza with laryngitis |
| 16388 | Influenza NOS |
| 17185 | Acute bronchiolitis with bronchospasm |
| 17359 | Chest infection - unspecified bronchitis |
| 17917 | Acute bronchiolitis NOS |
| 18451 | Acute bronchiolitis due to respiratory syncytial virus |
| 20198 | Acute bronchitis NOS |
| 21061 | Chronic obstruct pulmonary dis with acute lower resp infectn |
| 21145 | Acute croupous bronchitis |
| 21492 | Acute haemophilus influenzae bronchitis |
| 23488 | Influenza with respiratory manifestations NOS |
| 24316 | Chest infection with infectious disease EC |
| 24800 | Acute bacterial bronchitis unspecified |
| 26125 | Bronchiolitis obliterans |
| 29273 | Acute bronchitis due to parainfluenza virus |
| 29617 | Influenza with pharyngitis |
| 29669 | Acute bronchitis and bronchiolitis |
| 31363 | Influenza with other manifestations NOS |
| 37447 | Acute lower respiratory tract infection |
| 41137 | Acute bronchitis or bronchiolitis NOS |
| 41589 | Acute obliterating bronchiolitis |
| 43362 | Acute streptococcal bronchitis |
| 43625 | Influenza with other respiratory manifestation |
| 46157 | Influenza with encephalopathy |
| 47472 | Influenza with other manifestations |
| 48593 | Acute bronchitis due to respiratory syncytial virus |
| 49794 | Acute neisseria catarrhalis bronchitis |
| 54533 | Acute capillary bronchiolitis |
| 63697 | Avian influenza virus nucleic acid detection |
| 64890 | Acute bronchitis due to rhinovirus |
| 65916 | Acute bronchitis due to echovirus |
| 66228 | Acute bronchiolitis due to other specified organisms |
| 66397 | [X]Other acute lower respiratory infections |
| 69192 | Acute exudative bronchiolitis |
| 71370 | Acute pseudomembranous bronchitis |
| 73100 | [X]Acute bronchitis due to other specified organisms |
| 91123 | Parainfluenza type 3 nucleic acid detection |
| 93153 | Acute bronchitis due to coxsackievirus |
| 94130 | Parainfluenza type 1 nucleic acid detection |
| 94858 | Parainfluenza type 2 nucleic acid detection |
| 94930 | Avian influenza |
| 96017 | Influenza B virus detected |
| 96018 | Influenza H3 virus detected |
| 96019 | Influenza H1 virus detected |
| 96286 | Human parainfluenza virus detected |
| 97062 | Influenza A virus, other or untyped strain detected |
| 97279 | [X]Influenza+other manifestations, virus not identified |
| 97605 | [X]Influenza+oth respiratory manifestatns,virus not identifd |
| 97936 | [X]Influenza+other manifestations,influenza virus identified |
| 98102 | Influenza A (H1N1) swine flu |
| 98103 | Possible influenza A virus H1N1 subtype |
| 98115 | Suspected swine influenza |
| 98125 | Suspected influenza A virus subtype H1N1 infection |
| 98129 | Influenza due to Influenza A virus subtype H1N1 |
| 98143 | Influenza A virus H1N1 subtype detected |
| 98156 | Influenza H5 virus detected |
| 98257 | [X]Flu+oth respiratory manifestations,'flu virus identified |
| 99214 | [X]Acute bronchiolitis due to other specified organisms |
| 101775 | Acute membranous bronchitis |
| 102918 | Influenza H2 virus detected |

## Cough

| **Medical code** | **Read term** |
| --- | --- |
| 92 | Cough |
| 292 | Chesty cough |
| 1025 | Bronchial cough |
| 1160 | [D]Cough |
| 1234 | Productive cough NOS |
| 1273 | C/O - cough |
| 3068 | Night cough present |
| 3645 | Coughing up phlegm |
| 4070 | Morning cough |
| 4836 | Nocturnal cough / wheeze |
| 4931 | Dry cough |
| 7706 | Productive cough -clear sputum |
| 7707 | Cough symptom NOS |
| 7708 | Productive cough-yellow sputum |
| 7773 | Productive cough -green sputum |
| 8239 | [D]Cough with haemorrhage |
| 18907 | Cough with fever |
| 22318 | Difficulty in coughing up sputum |
| 29318 | Evening cough |
| 60903 | Cough aggravates symptom |
| 100515 | Cough swab |

## Breathlessness

| **Medical code** | **Read term** |
| --- | --- |
| 735 | [D]Breathlessness |
| 741 | [D]Shortness of breath |
| 1429 | Breathlessness |
| 2563 | [D]Respiratory distress |
| 2575 | Short of breath on exertion |
| 2737 | Respiratory distress syndrome |
| 2931 | Difficulty breathing |
| 3092 | [D]Dyspnoea |
| 4822 | Shortness of breath |
| 5175 | Breathlessness symptom |
| 5349 | Shortness of breath symptom |
| 5896 | Dyspnoea - symptom |
| 6326 | Breathless - moderate exertion |
| 6434 | Paroxysmal nocturnal dyspnoea |
| 7000 | O/E - dyspnoea |
| 7534 | O/E - respiratory distress |
| 7683 | Breathless - lying flat |
| 7932 | Breathless - mild exertion |
| 9297 | [D]Respiratory insufficiency |
| 18116 | Nocturnal dyspnoea |
| 21801 | Breathlessness NOS |
| 22094 | Short of breath dressing/undressing |
| 24889 | Breathless - strenuous exertion |
| 31143 | Breathless - at rest |
| 40813 | Unable to complete a sentence in one breath |
| 53771 | Dyspnoea on exertion |

## Sputum

| **Medical code** | **Read term** |
| --- | --- |
| 292 | Chesty cough |
| 1025 | Bronchial cough |
| 1234 | Productive cough NOS |
| 1251 | [D]Abnormal sputum |
| 3645 | Coughing up phlegm |
| 3727 | Sputum sent for C/S |
| 7706 | Productive cough -clear sputum |
| 7708 | Productive cough-yellow sputum |
| 7773 | Productive cough -green sputum |
| 8287 | Sputum sample obtained |
| 8760 | [D]Positive culture findings in sputum |
| 9807 | Sputum - symptom |
| 11072 | Acute purulent bronchitis |
| 14271 | Sputum culture |
| 14272 | Sputum microscopy |
| 14273 | Sputum appearance |
| 14804 | Sputum appears infected |
| 15430 | [D]Sputum abnormal - colour |
| 16026 | Sputum examination: abnormal |
| 18964 | Sputum clearance |
| 20086 | [D]Sputum abnormal - amount |
| 22318 | Difficulty in coughing up sputum |
| 23252 | Sputum microscopy NOS |
| 23582 | [D]Abnormal sputum NOS |
| 24181 | Sputum: mucopurulent |
| 30754 | Yellow sputum |
| 30904 | Sputum sent for examination |
| 36515 | [D]Abnormal sputum - tenacious |
| 36880 | Green sputum |
| 43270 | Sputum evidence of infection |
| 44214 | [D]Sputum abnormal - odour |
| 49144 | Sputum: pus cells present |
| 49694 | Sputum: organism on gram stain |
| 54177 | Sputum: excessive - mucoid |
| 100484 | Volume of sputum |
| 100524 | Moderate sputum |
| 100629 | White sputum |
| 100647 | Copious sputum |
| 100931 | Brown sputum |
| 101782 | Profuse sputum |
| 103209 | Grey sputum |

## Asthma

| **Medical code** | **Read term** |
| --- | --- |
| 78 | Asthma |
| 185 | Acute exacerbation of asthma |
| 13064 | Asthma severity |
| 16070 | Asthma NOS |
| 232 | Asthma attack |
| 10318 | Suspected asthma |
| 1555 | Bronchial asthma |
| 4442 | Asthma unspecified |
| 233 | Severe asthma attack |
| 7058 | Emergency admission asthma |
| 9018 | Number of asthma exacerbations in past year |
| 7146 | Extrinsic (atopic) asthma |
| 1208 | Childhood asthma |
| 3018 | Mild asthma |
| 13066 | Asthma - currently dormant |
| 2290 | Allergic asthma |
| 13065 | Moderate asthma |
| 10487 | Asthma - currently active |
| 3458 | Occasional asthma |
| 11370 | Asthma confirmed |
| 5267 | Intrinsic asthma |
| 3665 | Late onset asthma |
| 4892 | Status asthmaticus NOS |
| 6707 | Extrinsic asthma with asthma attack |
| 12987 | Late-onset asthma |
| 14777 | Extrinsic asthma without status asthmaticus |
| 3366 | Severe asthma |
| 8335 | Asthma attack NOS |
| 5798 | Chronic asthmatic bronchitis |
| 45782 | Extrinsic asthma NOS |
| 25796 | Mixed asthma |
| 40823 | Brittle asthma |
| 22752 | Occupational asthma |
| 23481 | Asthma - cardiac |
| 29325 | Intrinsic asthma without status asthmaticus |
| 45073 | Intrinsic asthma NOS |
| 27926 | Extrinsic asthma with status asthmaticus |
| 18323 | Intrinsic asthma with asthma attack |
| 21232 | Allergic asthma NEC |
| 58196 | Intrinsic asthma with status asthmaticus |
| 73522 | Work aggravated asthma |
| 39478 | Wood asthma |
| 40864 | [X] Adverse reaction to theophylline - asthma |
| 93353 | Sequoiosis (red-cedar asthma) |
| 26501 | Asthma never causes daytime symptoms |
| 26503 | Asthma causes daytime symptoms most days |
| 31225 | Asthma causes daytime symptoms 1 to 2 times per month |
| 24884 | Asthma causes daytime symptoms 1 to 2 times per week |
| 42824 | Asthma daytime symptoms |
| 31167 | Asthma night-time symptoms |
| 30815 | Asthma causing night waking |
| 39570 | Asthma causes night symptoms 1 to 2 times per month |
| 102400 | Asthma causes night time symptoms 1 to 2 times per week |
| 102395 | Asthma causes symptoms most nights |
| 103612 | Asthma never causes night symptoms |
| 13173 | Asthma not disturbing sleep |
| 13174 | Asthma not limiting activities |
| 26504 | Asthma never restricts exercise |
| 38143 | Asthma never disturbs sleep |
| 26861 | Asthma sometimes restricts exercise |
| 7416 | Asthma disturbing sleep |
| 7191 | Asthma limiting activities |
| 38144 | Asthma limits walking up hills or stairs |
| 25181 | Asthma restricts exercise |
| 13175 | Asthma disturbs sleep frequently |
| 38146 | Asthma disturbs sleep weekly |
| 26506 | Asthma severely restricts exercise |
| 38145 | Asthma limits walking on the flat |
| 103998 | Asthma limits activities most days |
| 102713 | Asthma limits activities 1 to 2 times per month |
| 102888 | Asthma limits activities 1 to 2 times per week |
| 13176 | Asthma follow-up |
| 719 | H/O: asthma |
| 19520 | Asthma treatment compliance satisfactory |
| 41020 | Absent from work or school due to asthma |
| 100509 | Under care of asthma specialist nurse |
| 5627 | Hay fever with asthma |
| 15248 | Hay fever with asthma |
| 11022 | Asthma trigger |
| 5867 | Exercise induced asthma |
| 102449 | Asthma trigger - respiratory infection |
| 102341 | Asthma trigger - pollen |
| 4606 | Exercise induced asthma |
| 103813 | Asthma trigger - cold air |
| 102871 | Asthma trigger - exercise |
| 103944 | Asthma trigger - airborne dust |
| 103321 | Asthma trigger - animals |
| 102301 | Asthma trigger - seasonal |
| 7731 | Pollen asthma |
| 103945 | Asthma trigger - damp |
| 102952 | Asthma trigger - warm air |
| 103952 | Asthma trigger - emotion |
| 103955 | Asthma trigger - tobacco smoke |
| 41017 | Aspirin induced asthma |
| 47684 | Detergent asthma |
| 47337 | Asthma accident and emergency attendance since last visit |
| 24479 | Emergency asthma admission since last appointment |
| 16667 | Asthma control step 2 |
| 18224 | Asthma control step 3 |
| 16785 | Asthma control step 1 |
| 29645 | Asthma control step 0 |
| 98185 | Asthma control test |
| 20886 | Asthma control step 4 |
| 20860 | Asthma control step 5 |
| 100397 | Asthma control questionnaire |

| **ICD10** | **Term** |
| --- | --- |
| J45 | Asthma |
| J45.1 | Non-allergic asthma |
| J45.8 | Mixed asthma |
| J45.9 | Asthma, unspecified |
| J46 | Status asthmaticus |

# Treatments

## LABA

| **prodcode** | **productname** |
| --- | --- |
| 1974 | Oxis 12 Turbohaler (AstraZeneca UK Ltd) |
| 1975 | Oxis 6 Turbohaler (AstraZeneca UK Ltd) |
| 6526 | Formoterol 12microgram inhalation powder capsules with device |
| 7133 | Formoterol 12micrograms/dose dry powder inhaler |
| 9711 | Formoterol 6micrograms/dose dry powder inhaler |
| 10968 | Foradil 12microgram inhalation powder capsules with device (Novartis Pharmaceuticals UK Ltd) |
| 14306 | Formoterol 12micrograms/dose inhaler CFC free |
| 25784 | Atimos Modulite 12micrograms/dose inhaler (Chiesi Ltd) |
| 35725 | Formoterol Easyhaler 12micrograms/dose dry powder inhaler (Orion Pharma (UK) Ltd) |
| 56482 | Oxis 12 Turbohaler (Waymade Healthcare Plc) |
| 43738 | Indacaterol 150microgram inhalation powder capsules with device |
| 43893 | Onbrez Breezhaler 150microgram inhalation powder capsules with device (Novartis Pharmaceuticals UK Ltd) |
| 44064 | Onbrez Breezhaler 300microgram inhalation powder capsules with device (Novartis Pharmaceuticals UK Ltd) |
| 45610 | Indacaterol 300microgram inhalation powder capsules with device |
| 62662 | Olodaterol 2.5micrograms/dose solution for inhalation cartridge with device CFC free |
| 465 | Salmeterol 25micrograms/dose inhaler |
| 549 | Serevent 25micrograms/dose inhaler (GlaxoSmithKline UK Ltd) |
| 719 | Salmeterol 50micrograms/dose dry powder inhaler |
| 910 | Serevent diskhaler 50microgram Inhalation powder (Glaxo Wellcome UK Ltd) |
| 2224 | Serevent 50micrograms/dose Accuhaler (GlaxoSmithKline UK Ltd) |
| 3297 | Salmeterol 50micrograms disc |
| 5558 | Salmeterol 50micrograms with fluticasone 500micrograms CFC free inhaler |
| 5864 | Salmeterol 25micrograms with fluticasone 250micrograms CFC free inhaler |
| 5942 | Salmeterol 50micrograms with fluticasone 250micrograms CFC free inhaler |
| 6569 | Salmeterol 25micrograms with fluticasone 125micrograms CFC free inhaler |
| 6616 | Salmeterol 25micrograms with fluticasone 50micrograms CFC free inhaler |
| 6938 | Salmeterol 50micrograms with fluticasone 100micrograms dry powder inhaler |
| 7268 | Serevent 25micrograms/dose Evohaler (GlaxoSmithKline UK Ltd) |
| 7270 | Salmeterol 25micrograms/dose inhaler CFC free |
| 35165 | Serevent 50microgram disks with Diskhaler (GlaxoSmithKline UK Ltd) |
| 35503 | Salmeterol 50microgram inhalation powder blisters |
| 35542 | Salmeterol 50microgram inhalation powder blisters with device |
| 35825 | Serevent 50microgram disks (GlaxoSmithKline UK Ltd) |
| 47638 | Neovent 25micrograms/dose inhaler CFC free (Fannin UK Ltd) |
| 50051 | Serevent 25micrograms/dose Evohaler (Waymade Healthcare Plc) |
| 54742 | Salmeterol 25micrograms/dose inhaler CFC free (A A H Pharmaceuticals Ltd) |
| 56478 | Serevent 50micrograms/dose Accuhaler (Doncaster Pharmaceuticals Ltd) |

## LAMA

| **prodcode** | **productname** |
| --- | --- |
| 49227 | Aclidinium bromide 375micrograms/dose dry powder inhaler |
| 49228 | Eklira 322micrograms/dose Genuair (Almirall Ltd) |
| 63992 | Eklira 322micrograms/dose Genuair (Waymade Healthcare Plc) |
| 53761 | Glycopyrronium bromide 55microgram inhalation powder capsules with device |
| 53982 | Seebri Breezhaler 44microgram inhalation powder capsules with device (Novartis Pharmaceuticals UK Ltd) |
| 36869 | Spiriva Respimat 2.5micrograms/dose solution for inhalation cartridge with device (Boehringer Ingelheim Ltd) |
| 61582 | Spiriva Respimat 2.5micrograms/dose solution for inhalation cartridge with device (Waymade Healthcare Plc) |
| 746 | Tiotropium 18 microgram Capsule |
| 6050 | Spiriva 18 microgram Capsule (Boehringer Ingelheim Ltd) |
| 34995 | Spiriva 18microgram inhalation powder capsules with HandiHaler (Boehringer Ingelheim Ltd) |
| 35000 | Spiriva 18microgram inhalation powder capsules (Boehringer Ingelheim Ltd) |
| 50577 | Spiriva 18microgram inhalation powder capsules with HandiHaler (DE Pharmaceuticals) |
| 51967 | Spiriva 18microgram inhalation powder capsules (Mawdsley-Brooks & Company Ltd) |
| 36864 | Tiotropium bromide 2.5micrograms/dose solution for inhalation cartridge with device CFC free |
| 64232 | Tiotropium bromide 2.5micrograms/dose solution for inhalation cartridge with device CFC free (AM Distributions (Yorkshire) Ltd) |
| 35011 | Tiotropium bromide 18microgram inhalation powder capsules |
| 35014 | Tiotropium bromide 18microgram inhalation powder capsules with device |
| 62109 | Umeclidinium bromide 65microgram/dose/dry powder inhaler |

## Inhaled corticosteroid

| **prodcode** | **productname** |
| --- | --- |
| 38 | Beclometasone 100micrograms/dose inhaler |
| 99 | Becotide 100 inhaler (GlaxoSmithKline UK Ltd) |
| 883 | Becodisks 200microgram Disc (Allen & Hanburys Ltd) |
| 895 | Beclazone 100 Easi-Breathe inhaler (Teva UK Ltd) |
| 896 | Becotide easi-breathe 100microgram/actuation Pressurised inhalation (Allen & Hanburys Ltd) |
| 1100 | Beclazone 100 inhaler (Teva UK Ltd) |
| 1236 | Becloforte 250micrograms/dose inhaler (GlaxoSmithKline UK Ltd) |
| 1242 | Beclometasone 250micrograms/dose inhaler |
| 1243 | Beclazone 250 Easi-Breathe inhaler (Teva UK Ltd) |
| 1258 | Becotide 200 inhaler (GlaxoSmithKline UK Ltd) |
| 1259 | Beclometasone 200micrograms/dose inhaler |
| 1406 | Becotide 50 inhaler (GlaxoSmithKline UK Ltd) |
| 1537 | Becotide 200microgram Rotacaps (GlaxoSmithKline UK Ltd) |
| 1551 | Beclazone 250 inhaler (Teva UK Ltd) |
| 1552 | Becloforte easi-breathe 250microgram/actuation Pressurised inhalation (Allen & Hanburys Ltd) |
| 1725 | Beclazone 50 Easi-Breathe inhaler (Teva UK Ltd) |
| 1727 | Becotide easi-breathe 50microgram/actuation Pressurised inhalation (Allen & Hanburys Ltd) |
| 1734 | Beclometasone 100micrograms/dose breath actuated inhaler |
| 1861 | AeroBec 100 Autohaler (Meda Pharmaceuticals Ltd) |
| 1885 | Beclazone 200 inhaler (Teva UK Ltd) |
| 1951 | Becodisks 400microgram Disc (Allen & Hanburys Ltd) |
| 2148 | Beclometasone 400microgram disc |
| 2159 | AeroBec 50 Autohaler (Meda Pharmaceuticals Ltd) |
| 2160 | Beclometasone 50micrograms/dose breath actuated inhaler |
| 2229 | Becodisks 100microgram Disc (Allen & Hanburys Ltd) |
| 2335 | Qvar 100 inhaler (Teva UK Ltd) |
| 2600 | Beclometasone 250micrograms/dose breath actuated inhaler |
| 2892 | Becloforte 400microgram disks (GlaxoSmithKline UK Ltd) |
| 2893 | Beclometasone 200micrograms disc |
| 2992 | Beclazone 50 inhaler (Teva UK Ltd) |
| 3018 | Beclometasone 50micrograms/dose inhaler |
| 3075 | Becotide 400microgram Rotacaps (GlaxoSmithKline UK Ltd) |
| 3119 | Becloforte integra 250microgram/actuation Inhaler with compact spacer (Glaxo Laboratories Ltd) |
| 3150 | Beclometasone 100micrograms/actuation extrafine particle cfc free inhaler |
| 3220 | Qvar 50 Autohaler (Teva UK Ltd) |
| 3363 | Becloforte 400microgram disks with Diskhaler (GlaxoSmithKline UK Ltd) |
| 3546 | Qvar 50 inhaler (Teva UK Ltd) |
| 3556 | Beclometasone 50micrograms with salbutamol 100micrograms/inhalation inhaler |
| 3743 | Filair 50 inhaler (Meda Pharmaceuticals Ltd) |
| 3927 | Filair 100 inhaler (Meda Pharmaceuticals Ltd) |
| 3947 | Becotide 100microgram Rotacaps (GlaxoSmithKline UK Ltd) |
| 3993 | Filair Forte 250micrograms/dose inhaler (Meda Pharmaceuticals Ltd) |
| 4365 | Beclometasone 100micrograms disc |
| 4413 | Qvar 100 Autohaler (Teva UK Ltd) |
| 4499 | Aerobec 250microgram/actuation Pressurised inhalation (Meda Pharmaceuticals Ltd) |
| 4601 | Asmabec 100 Clickhaler (Focus Pharmaceuticals Ltd) |
| 4759 | Beclometasone 100microgram inhalation powder capsules |
| 4803 | Beclazone 250microgram/actuation Inhalation powder (Actavis UK Ltd) |
| 5521 | Beclometasone 200micrograms/dose dry powder inhaler |
| 5522 | Beclometasone 100micrograms/dose dry powder inhaler |
| 5804 | Beclometasone 250micrograms/dose dry powder inhaler |
| 5992 | Beclometasone 50micrograms/dose dry powder inhaler |
| 7653 | Beclometasone 400microgram inhalation powder capsules |
| 8111 | Becloforte vm 250microgram/actuation VM pack (Allen & Hanburys Ltd) |
| 9233 | Beclometasone 200microgram inhalation powder capsules |
| 9477 | Asmabec 100microgram/actuation Spacehaler (Celltech Pharma Europe Ltd) |
| 9571 | Beclometasone 250micrograms/actuation vortex inhaler |
| 9577 | Asmabec 50 Clickhaler (Focus Pharmaceuticals Ltd) |
| 9599 | Beclazone 50microgram/actuation Inhalation powder (Actavis UK Ltd) |
| 9921 | Beclometasone 100micrograms/dose breath actuated inhaler CFC free |
| 10090 | Beclometasone 50micrograms/actuation extrafine particle cfc free inhaler |
| 11198 | Beclometasons 50 micrograms/actuation vortex inhaler |
| 11497 | Beclometasone 400micrograms/dose dry powder inhaler |
| 11732 | Beclometasone 50micrograms/dose breath actuated inhaler CFC free |
| 13037 | Pulvinal Beclometasone Dipropionate 200micrograms/dose dry powder inhaler (Chiesi Ltd) |
| 13290 | Clenil Modulite 100micrograms/dose inhaler (Chiesi Ltd) |
| 13815 | Beclazone 100microgram/actuation Inhalation powder (Actavis UK Ltd) |
| 14294 | Qvar 50micrograms/dose Easi-Breathe inhaler (Teva UK Ltd) |
| 14321 | Beclometasone 200micrograms/dose inhaler CFC free |
| 14524 | Bdp 250microgram/actuation Spacehaler (Celltech Pharma Europe Ltd) |
| 14567 | Asmabec 250 Clickhaler (Focus Pharmaceuticals Ltd) |
| 14590 | Asmabec 250microgram/actuation Spacehaler (Celltech Pharma Europe Ltd) |
| 14736 | Pulvinal Beclometasone Dipropionate 400micrograms/dose dry powder inhaler (Chiesi Ltd) |
| 14757 | Pulvinal Beclometasone Dipropionate 100micrograms/dose dry powder inhaler (Chiesi Ltd) |
| 15326 | Beclometasone 100micrograms/dose inhaler CFC free |
| 15706 | Beclometasone 100 micrograms/actuation vortex inhaler |
| 16148 | Clenil Modulite 250micrograms/dose inhaler (Chiesi Ltd) |
| 16151 | Clenil Modulite 200micrograms/dose inhaler (Chiesi Ltd) |
| 16158 | Clenil Modulite 50micrograms/dose inhaler (Chiesi Ltd) |
| 16584 | Beclometasone 50micrograms/dose inhaler CFC free |
| 17654 | Easyhaler Beclometasone 200micrograms/dose dry powder inhaler (Orion Pharma (UK) Ltd) |
| 18394 | Bdp 50microgram/actuation Spacehaler (Celltech Pharma Europe Ltd) |
| 18848 | Qvar 100micrograms/dose Easi-Breathe inhaler (Teva UK Ltd) |
| 19031 | Bdp 100microgram/actuation Spacehaler (Celltech Pharma Europe Ltd) |
| 19121 | Beclometasone 100micrograms with Salbutamol 200micrograms inhalation capsules |
| 19376 | Beclometasone 200micrograms with Salbutamol 400micrograms inhalation capsules |
| 19389 | Asmabec 50microgram/actuation Spacehaler (Celltech Pharma Europe Ltd) |
| 19401 | Beclometasone 250micrograms/actuation inhaler and compact spacer |
| 20825 | Spacehaler BDP 250microgram/actuation Spacehaler (Celltech Pharma Europe Ltd) |
| 21005 | Beclometasone 250micrograms/dose inhaler CFC free |
| 21482 | Beclometasone 100micrograms/dose inhaler (Generics (UK) Ltd) |
| 24898 | Spacehaler BDP 100microgram/actuation Spacehaler (Celltech Pharma Europe Ltd) |
| 25204 | Beclometasone 100micrograms/dose inhaler (A A H Pharmaceuticals Ltd) |
| 26063 | Beclometasone 100micrograms/dose inhaler (Teva UK Ltd) |
| 27679 | Beclometasone 100microgram/actuation Pressurised inhalation (Approved Prescription Services Ltd) |
| 28073 | Beclometasone 250microgram/actuation Pressurised inhalation (Approved Prescription Services Ltd) |
| 28640 | Beclometasone 100microgram/actuation Inhalation powder (Actavis UK Ltd) |
| 28761 | Spacehaler BDP 50microgram/actuation Spacehaler (Celltech Pharma Europe Ltd) |
| 29325 | Beclometasone 250micrograms/dose inhaler (Generics (UK) Ltd) |
| 30210 | Beclometasone 250micrograms/dose inhaler (Teva UK Ltd) |
| 30238 | Beclometasone 50microgram/actuation Pressurised inhalation (Approved Prescription Services Ltd) |
| 31774 | Beclometasone 50micrograms/dose inhaler (Generics (UK) Ltd) |
| 32874 | Beclometasone 50microgram/actuation Inhalation powder (Actavis UK Ltd) |
| 33258 | Beclometasone 250micrograms/dose inhaler (A A H Pharmaceuticals Ltd) |
| 33849 | Beclometasone 100microgram/actuation Inhalation powder (Neo Laboratories Ltd) |
| 34315 | Beclometasone 250microgram/actuation Inhalation powder (Actavis UK Ltd) |
| 34428 | Beclometasone 50microgram/actuation Inhalation powder (Neo Laboratories Ltd) |
| 34739 | Beclometasone 50micrograms/dose inhaler (Teva UK Ltd) |
| 34794 | Beclometasone 200micrograms/dose inhaler (A A H Pharmaceuticals Ltd) |
| 34859 | Beclometasone 250microgram/actuation Inhalation powder (Neo Laboratories Ltd) |
| 34919 | Beclometasone 50micrograms/dose inhaler (A A H Pharmaceuticals Ltd) |
| 35071 | Becodisks 200microgram (GlaxoSmithKline UK Ltd) |
| 35106 | Becodisks 100microgram with Diskhaler (GlaxoSmithKline UK Ltd) |
| 35107 | Beclometasone 400microgram inhalation powder blisters with device |
| 35113 | Beclometasone 200microgram inhalation powder blisters |
| 35118 | Becodisks 400microgram with Diskhaler (GlaxoSmithKline UK Ltd) |
| 35288 | Beclometasone 400microgram inhalation powder blisters |
| 35293 | Beclometasone 200microgram inhalation powder blisters with device |
| 35299 | Becodisks 400microgram (GlaxoSmithKline UK Ltd) |
| 35408 | Becodisks 100microgram (GlaxoSmithKline UK Ltd) |
| 35430 | Becodisks 200microgram with Diskhaler (GlaxoSmithKline UK Ltd) |
| 35580 | Beclometasone 100microgram inhalation powder blisters with device |
| 35652 | Beclometasone 100microgram inhalation powder blisters |
| 39200 | AeroBec Forte 250 Autohaler (Meda Pharmaceuticals Ltd) |
| 41269 | Beclometasone 400 Cyclocaps (Teva UK Ltd) |
| 41412 | Beclometasone 400micrograms/actuation inhaler |
| 46157 | Beclometasone 200 Cyclocaps (Teva UK Ltd) |
| 47943 | Beclazone easi-breathe (roi) 100microgram/actuation Pressurised inhalation (Ivax Pharmaceuticals Ireland) |
| 48340 | Clenil Modulite 100micrograms/dose inhaler (Mawdsley-Brooks & Company Ltd) |
| 48709 | Qvar 100micrograms/dose Easi-Breathe inhaler (Sigma Pharmaceuticals Plc) |
| 49367 | Clenil Modulite 50micrograms/dose inhaler (Mawdsley-Brooks & Company Ltd) |
| 49412 | Clenil Modulite 200micrograms/dose inhaler (Mawdsley-Brooks & Company Ltd) |
| 50129 | Qvar 100micrograms/dose Easi-Breathe inhaler (Doncaster Pharmaceuticals Ltd) |
| 50287 | Qvar 100 inhaler (Doncaster Pharmaceuticals Ltd) |
| 51234 | Qvar 100 inhaler (Waymade Healthcare Plc) |
| 51415 | Qvar 50 inhaler (Mawdsley-Brooks & Company Ltd) |
| 51480 | Qvar 100 Autohaler (Doncaster Pharmaceuticals Ltd) |
| 51681 | Qvar 100 inhaler (Sigma Pharmaceuticals Plc) |
| 52806 | Qvar 100 Autohaler (Lexon (UK) Ltd) |
| 53480 | Qvar 100 Autohaler (Stephar (U.K.) Ltd) |
| 54207 | Qvar 50 inhaler (Doncaster Pharmaceuticals Ltd) |
| 54399 | Qvar 100 Autohaler (Sigma Pharmaceuticals Plc) |
| 56462 | Becodisks 400microgram (Waymade Healthcare Plc) |
| 56471 | Becodisks 200microgram (Mawdsley-Brooks & Company Ltd) |
| 56493 | Qvar 50micrograms/dose Easi-Breathe inhaler (Sigma Pharmaceuticals Plc) |
| 57589 | Becloforte 250micrograms/dose inhaler (Dowelhurst Ltd) |
| 1269 | Becotide 50microgram/ml Nebuliser liquid (Allen & Hanburys Ltd) |
| 7964 | Beclometasone 50micrograms/ml nebuliser suspension |
| 3065 | Bextasol Inhalation powder (Allen & Hanburys Ltd) |
| 7724 | Betamethasone valerate 100micrograms/actuation inhaler |
| 454 | Pulmicort 200microgram Inhaler (AstraZeneca UK Ltd) |
| 908 | Pulmicort 400 Turbohaler (AstraZeneca UK Ltd) |
| 909 | Budesonide 200micrograms/dose inhaler |
| 947 | Budesonide 50micrograms/actuation refill canister |
| 956 | Pulmicort 200 Turbohaler (AstraZeneca UK Ltd) |
| 959 | Budesonide 50micrograms/dose inhaler |
| 960 | Pulmicort 100 Turbohaler (AstraZeneca UK Ltd) |
| 1642 | Budesonide 400micrograms/dose dry powder inhaler |
| 1680 | Pulmicort LS 50micrograms/dose inhaler (AstraZeneca UK Ltd) |
| 1956 | Pulmicort 1mg Respules (AstraZeneca UK Ltd) |
| 1959 | Pulmicort 0.5mg Respules (AstraZeneca UK Ltd) |
| 2092 | Budesonide 200micrograms/dose dry powder inhaler |
| 2125 | Pulmicort 200microgram Refill canister (AstraZeneca UK Ltd) |
| 3570 | Budesonide 200micrograms/actuation refill canister |
| 4545 | Pulmicort LS 50microgram Refill canister (AstraZeneca UK Ltd) |
| 4801 | Budesonide 500micrograms/2ml nebuliser liquid unit dose vials |
| 4942 | Budesonide 1mg/2ml nebuliser liquid unit dose vials |
| 7788 | Budesonide 100micrograms/dose dry powder inhaler |
| 8433 | Budesonide 100micrograms/actuation inhaler |
| 10321 | Budesonide 400microgram inhalation powder capsules |
| 14700 | Budesonide 400micrograms/actuation inhaler |
| 16054 | Budesonide 200micrograms/actuation breath actuated powder inhaler |
| 17670 | Easyhaler Budesonide 100micrograms/dose dry powder inhaler (Orion Pharma (UK) Ltd) |
| 18537 | Budesonide 200microgram inhalation powder capsules |
| 23741 | Novolizer budesonide 200microgram/actuation Pressurised inhalation (Meda Pharmaceuticals Ltd) |
| 27188 | Easyhaler Budesonide 200micrograms/dose dry powder inhaler (Orion Pharma (UK) Ltd) |
| 30649 | Easyhaler Budesonide 400micrograms/dose dry powder inhaler (Orion Pharma (UK) Ltd) |
| 35510 | Budesonide 200micrograms/dose dry powder inhalation cartridge with device |
| 35602 | Budesonide 200micrograms/dose dry powder inhalation cartridge |
| 35631 | Budelin Novolizer 200micrograms/dose inhalation powder (Meda Pharmaceuticals Ltd) |
| 35724 | Budelin Novolizer 200micrograms/dose inhalation powder refill (Meda Pharmaceuticals Ltd) |
| 39099 | Pulmicort 100micrograms/dose inhaler CFC free (AstraZeneca UK Ltd) |
| 39102 | Budesonide 100micrograms/dose inhaler CFC free |
| 39879 | Budesonide 200micrograms/dose inhaler CFC free |
| 40057 | Pulmicort 200micrograms/dose inhaler CFC free (AstraZeneca UK Ltd) |
| 49711 | Pulmicort 200micrograms/dose inhaler (AstraZeneca UK Ltd) |
| 50037 | Pulmicort 0.5mg Respules (Waymade Healthcare Plc) |
| 52732 | Pulmicort 0.5mg Respules (Necessity Supplies Ltd) |
| 56498 | Pulmicort 200 Turbohaler (Waymade Healthcare Plc) |
| 6839 | Alvesco 160 inhaler (Takeda UK Ltd) |
| 7356 | Ciclesonide 80micrograms/dose inhaler CFC free |
| 10102 | Ciclesonide 160micrograms/dose inhaler CFC free |
| 21224 | Alvesco 80 inhaler (Takeda UK Ltd) |
| 911 | Flixotide accuhaler 250 250microgram/inhalation Inhalation powder (Allen & Hanburys Ltd) |
| 1412 | Flixotide 250microgram/actuation Inhalation powder (Allen & Hanburys Ltd) |
| 1424 | Flixotide 250microgram Disc (Allen & Hanburys Ltd) |
| 1426 | Flixotide 500microgram Disc (Allen & Hanburys Ltd) |
| 1518 | Flixotide 50microgram/actuation Inhalation powder (Allen & Hanburys Ltd) |
| 1676 | Flixotide 125microgram/actuation Inhalation powder (Allen & Hanburys Ltd) |
| 2282 | Fluticasone 500micrograms/dose dry powder inhaler |
| 2440 | Flixotide accuhaler 500 500microgram/inhalation Inhalation powder (Allen & Hanburys Ltd) |
| 2723 | Fluticasone 25micrograms/dose inhaler |
| 2951 | Fluticasone 250microgram/actuation Pressurised inhalation |
| 3289 | Flixotide 25micrograms/dose inhaler (GlaxoSmithKline UK Ltd) |
| 3989 | Flixotide 100microgram Disc (Allen & Hanburys Ltd) |
| 4131 | Fluticasone 100microgram Disc |
| 4132 | Fluticasone 125microgram/actuation Pressurised inhalation |
| 4688 | Fluticasone 50microgram/actuation Pressurised inhalation |
| 4926 | Flixotide accuhaler 100 100microgram/inhalation Inhalation powder (Allen & Hanburys Ltd) |
| 5223 | Fluticasone 50micrograms/dose inhaler CFC free |
| 5309 | Flixotide 50micrograms/dose Evohaler (GlaxoSmithKline UK Ltd) |
| 5551 | Flixotide 0.5mg/2ml Nebules (GlaxoSmithKline UK Ltd) |
| 5580 | Flixotide accuhaler 50 50microgram/inhalation Inhalation powder (Allen & Hanburys Ltd) |
| 5683 | Flixotide 250micrograms/dose Evohaler (GlaxoSmithKline UK Ltd) |
| 5718 | Flixotide 125micrograms/dose Evohaler (GlaxoSmithKline UK Ltd) |
| 5822 | Fluticasone 250micrograms/dose inhaler CFC free |
| 5885 | Fluticasone 100micrograms/dose dry powder inhaler |
| 5975 | Fluticasone 125micrograms/dose inhaler CFC free |
| 7602 | Fluticasone 50microgram Disc |
| 7638 | Fluticasone 250microgram Disc |
| 7891 | Fluticasone 500microgram Disc |
| 7948 | Fluticasone 250micrograms/dose dry powder inhaler |
| 8635 | Flixotide 50microgram Disc (Allen & Hanburys Ltd) |
| 9164 | Fluticasone 50micrograms/dose dry powder inhaler |
| 11478 | Fluticasone 2mg/2ml nebuliser liquid unit dose vials |
| 16305 | Flixotide 2mg/2ml Nebules (GlaxoSmithKline UK Ltd) |
| 17465 | Fluticasone 500micrograms/2ml nebuliser liquid unit dose vials |
| 35225 | Flixotide 100microgram disks with Diskhaler (GlaxoSmithKline UK Ltd) |
| 35374 | Flixotide 500microgram disks (GlaxoSmithKline UK Ltd) |
| 35392 | Flixotide 500microgram disks with Diskhaler (GlaxoSmithKline UK Ltd) |
| 35461 | Flixotide 250microgram disks with Diskhaler (GlaxoSmithKline UK Ltd) |
| 35611 | Flixotide 250microgram disks (GlaxoSmithKline UK Ltd) |
| 35638 | Fluticasone 100microgram inhalation powder blisters with device |
| 35700 | Fluticasone 500microgram inhalation powder blisters with device |
| 35772 | Fluticasone 100microgram inhalation powder blisters |
| 35905 | Fluticasone 250microgram inhalation powder blisters |
| 35986 | Flixotide 50microgram disks (GlaxoSmithKline UK Ltd) |
| 36021 | Fluticasone 50microgram inhalation powder blisters with device |
| 36090 | Flixotide 100microgram disks (GlaxoSmithKline UK Ltd) |
| 36290 | Flixotide 50microgram disks with Diskhaler (GlaxoSmithKline UK Ltd) |
| 36401 | Fluticasone 250microgram inhalation powder blisters with device |
| 36462 | Fluticasone 500microgram inhalation powder blisters |
| 37447 | Fluticasone 50microgram inhalation powder blisters |
| 42928 | Flixotide 100micrograms/dose Accuhaler (GlaxoSmithKline UK Ltd) |
| 42985 | Flixotide 50micrograms/dose Accuhaler (GlaxoSmithKline UK Ltd) |
| 42994 | Flixotide 250micrograms/dose Accuhaler (GlaxoSmithKline UK Ltd) |
| 43074 | Flixotide 500micrograms/dose Accuhaler (GlaxoSmithKline UK Ltd) |
| 49772 | Fluticasone 250micrograms/dose Evohaler (Sigma Pharmaceuticals Plc) |
| 51815 | Flixotide 250micrograms/dose Evohaler (Waymade Healthcare Plc) |
| 53057 | Flixotide 50micrograms/dose Evohaler (Lexon (UK) Ltd) |
| 56474 | Flixotide 125micrograms/dose Evohaler (Doncaster Pharmaceuticals Ltd) |
| 56475 | Flixotide 50micrograms/dose Accuhaler (Sigma Pharmaceuticals Plc) |
| 56477 | Flixotide 100micrograms/dose Accuhaler (Waymade Healthcare Plc) |
| 56484 | Flixotide 250micrograms/dose Accuhaler (Waymade Healthcare Plc) |
| 56499 | Flixotide 500micrograms/dose Accuhaler (Waymade Healthcare Plc) |
| 57525 | Flixotide 250micrograms/dose Accuhaler (Stephar (U.K.) Ltd) |
| 57555 | Flixotide 125micrograms/dose Evohaler (Dowelhurst Ltd) |
| 57579 | Flixotide 50micrograms/dose Accuhaler (Doncaster Pharmaceuticals Ltd) |
| 10254 | Mometasone 400micrograms/dose dry powder inhaler |
| 16018 | Mometasone 200micrograms/dose dry powder inhaler |
| 16433 | Asmanex 200micrograms/dose Twisthaler (Merck Sharp & Dohme Ltd) |
| 17590 | Asmanex 400micrograms/dose Twisthaler (Merck Sharp & Dohme Ltd) |

# Consultations

| **Medical code** | **Read term** | **Unplanned respiratory-related** | **Routine respiratory-related** |
| --- | --- | --- | --- |
| 3055 | Referred to chest physician | 1 | 0 |
| 1853 | [D]Chest lump | 1 | 0 |
| 3092 | [D]Dyspnoea | 1 | 0 |
| 9340 | [D]Non cardiac chest pain | 1 | 0 |
| 7844 | [D]Non-cardiac chest pain | 1 | 0 |
| 14819 | [D]Painful respiration NOS | 1 | 0 |
| 18183 | [D]Pleuritic pain | 1 | 0 |
| 103503 | [D]Pulmonary nodule | 1 | 0 |
| 3959 | [D]Respiratory failure | 1 | 0 |
| 101073 | [D]Severe wheeze | 1 | 0 |
| 741 | [D]Shortness of breath | 1 | 0 |
| 101421 | [D]Very severe wheeze | 1 | 0 |
| 2210 | [D]Wheezing | 1 | 0 |
| 1019 | Acute bronchiolitis | 1 | 0 |
| 312 | Acute bronchitis | 1 | 0 |
| 41137 | Acute bronchitis and bronchiolitis | 1 | 0 |
| 1446 | Acute exacerbation of chronic obstructive airway | 1 | 0 |
| 6124 | Acute lower respiratory tract infection | 1 | 0 |
| 37447 | Acute lower respiratory tract infection | 1 | 0 |
| 41589 | Acute obliterating bronchiolitis | 1 | 0 |
| 980 | Acute sinusitis | 1 | 0 |
| 1382 | Acute viral bronchitis unspecified | 1 | 0 |
| 5978 | Acute wheezy bronchitis | 1 | 0 |
| 11019 | Admit COPD emergency | 1 | 0 |
| 30134 | Adverse reaction to prednisolone | 1 | 0 |
| 100123 | Antibiotic therapy for acute pulmonary exacerbat | 1 | 0 |
| 10992 | Aspiration pneumonitis | 1 | 0 |
| 3683 | Basal pneumonia due to unspecified organism | 1 | 0 |
| 4135 | Blood in sputum - haemoptysis | 1 | 0 |
| 10013 | Blood in sputum - symptom | 1 | 0 |
| 98137 | Brief intervention for smoking cessation | 1 | 0 |
| 886 | Bronchopneumonia due to unspecified organism | 1 | 0 |
| 5654 | Bronchoscopy abnormal | 1 | 0 |
| 2870 | Bronchoscopy NEC | 1 | 0 |
| 3056 | Bronchoscopy normal | 1 | 0 |
| 9653 | Bronchospasm | 1 | 0 |
| 6348 | Bullae of lung | 1 | 0 |
| 6695 | C/O - a chest wall symptom | 1 | 0 |
| 2476 | Chest cold | 1 | 0 |
| 68 | Chest infection | 1 | 0 |
| 17359 | Chest infection - unspecified bronchitis | 1 | 0 |
| 16287 | Chest infection - unspecified bronchopneumonia | 1 | 0 |
| 2581 | Chest infection NOS | 1 | 0 |
| 2018 | Chest X-ray - routine | 1 | 0 |
| 7884 | Chron obstruct pulmonary dis wth acute exacerbat | 1 | 0 |
| 21061 | Chronic obstruct pulmonary dis with acute lower | 1 | 0 |
| 104998 | Chronic obstructve pulmonry disease rescue pack | 1 | 0 |
| 8370 | Collapse of lung | 1 | 0 |
| 104121 | Community acquired pneumonia | 1 | 0 |
| 85669 | Computed tomography of chest | 1 | 0 |
| 97218 | Computed tomography pulmonary angiography | 1 | 0 |
| 22418 | CPAP - Continuous positive airways pressure | 1 | 0 |
| 28444 | CPAP - Continuous positive airways pressure | 1 | 0 |
| 100962 | CT (computed tomography) of chest and abdomen | 1 | 0 |
| 22318 | Difficulty in coughing up sputum | 1 | 0 |
| 6051 | Diffuse pulmonary fibrosis | 1 | 0 |
| 7058 | Emergency admission, asthma | 1 | 0 |
| 2375 | Empyema | 1 | 0 |
| 2157 | Flu like illness | 1 | 0 |
| 36880 | Green sputum | 1 | 0 |
| 104264 | Hospital acquired pneumonia | 1 | 0 |
| 556 | Influenza | 1 | 0 |
| 5947 | Influenza like illness | 1 | 0 |
| 16388 | Influenza NOS | 1 | 0 |
| 8980 | Influenza-like symptoms | 1 | 0 |
| 4910 | Interstitial pneumonia | 1 | 0 |
| 1849 | Lobar (pneumococcal) pneumonia | 1 | 0 |
| 700 | Lobectomy of lung | 1 | 0 |
| 3358 | Lower resp tract infection | 1 | 0 |
| 3903 | Malignant neoplasm of bronchus or lung NOS | 1 | 0 |
| 33444 | Malignant neoplasm of hilus of lung | 1 | 0 |
| 18678 | Malignant neoplasm of lower lobe bronchus | 1 | 0 |
| 12582 | Malignant neoplasm of lower lobe of lung | 1 | 0 |
| 31188 | Malignant neoplasm of lower lobe, bronchus or lu | 1 | 0 |
| 12870 | Malignant neoplasm of main bronchus | 1 | 0 |
| 13243 | Malignant neoplasm of trachea, bronchus and lung | 1 | 0 |
| 25886 | Malignant neoplasm of upper lobe of lung | 1 | 0 |
| 10358 | Malignant neoplasm of upper lobe, bronchus or lu | 1 | 0 |
| 11849 | Other specified pneumonia or influenza | 1 | 0 |
| 26447 | Plain x-ray of chest | 1 | 0 |
| 9559 | Pleural effusion NOS | 1 | 0 |
| 947 | Pleural effusion NOS | 1 | 0 |
| 978 | Pleurisy | 1 | 0 |
| 1059 | Pleuritic pain | 1 | 0 |
| 572 | Pneumonia due to unspecified organism | 1 | 0 |
| 98103 | Possible influenza A virus H1N1 subtype | 1 | 0 |
| 1266 | Pulmonary embolism | 1 | 0 |
| 9701 | Pulmonary embolus | 1 | 0 |
| 32223 | Pulmonary mycobacterial infection | 1 | 0 |
| 635 | Pulmonary tuberculosis | 1 | 0 |
| 12509 | Referral to rapid access chest pain clinic | 1 | 0 |
| 48457 | Respiratory acidosis | 1 | 0 |
| 25249 | Respiratory failure | 1 | 0 |
| 293 | Respiratory tract infection | 1 | 0 |
| 3821 | Rhinitis - acute | 1 | 0 |
| 9714 | Seen in rapid access chest pain clinic | 1 | 0 |
| 243 | Sinusitis | 1 | 0 |
| 14804 | Sputum appears infected | 1 | 0 |
| 511 | Standard chest X-ray | 1 | 0 |
| 664 | Standard chest X-ray abnormal | 1 | 0 |
| 4247 | TB chemotherapy | 1 | 0 |
| 1840 | Tuberculosis | 1 | 0 |
| 2637 | Upper respiratory tract infection NOS | 1 | 0 |
| 2575 | Short of breath on exertion | 0 | 1 |
| 20107 | [D]Abnormal chest sounds | 0 | 1 |
| 735 | [D]Breathlessness | 0 | 1 |
| 1160 | [D]Cough | 0 | 1 |
| 5359 | [D]Pulmonary function studies abnormal | 0 | 1 |
| 2035 | [V]Routine chest X-ray | 0 | 1 |
| 41020 | Absent from work or school due to asthma | 0 | 1 |
| 185 | Acute exacerbation of asthma | 0 | 1 |
| 8025 | Acute respiratory infections | 0 | 1 |
| 6294 | Acute upper respiratory tract infection | 0 | 1 |
| 100459 | Advance supply of steroid medication | 0 | 1 |
| 9045 | Advice on smoking | 0 | 1 |
| 9177 | Airways obstruction reversible | 0 | 1 |
| 4084 | Airways obstructn irreversible | 0 | 1 |
| 2290 | Allergic asthma | 0 | 1 |
| 175 | Allergic rhinitis | 0 | 1 |
| 3019 | Alpha-1-antitrypsin deficiency | 0 | 1 |
| 28834 | Anti-smoking monitoring admin. | 0 | 1 |
| 101046 | Assessment for home oxygen therapy | 0 | 1 |
| 58151 | Assessment for pulmonary rehabilitation complete | 0 | 1 |
| 78 | Asthma | 0 | 1 |
| 13066 | Asthma - currently dormant | 0 | 1 |
| 47337 | Asthma accident and emergency attendance since l | 0 | 1 |
| 10043 | Asthma annual review | 0 | 1 |
| 232 | Asthma attack | 0 | 1 |
| 31225 | Asthma causes daytime symptoms 1 to 2 times per | 0 | 1 |
| 26503 | Asthma causes daytime symptoms most days | 0 | 1 |
| 39570 | Asthma causes night symptoms 1 to 2 times per mo | 0 | 1 |
| 102400 | Asthma causes night time symptoms 1 to 2 times p | 0 | 1 |
| 102395 | Asthma causes symptoms most nights | 0 | 1 |
| 30815 | Asthma causing night waking | 0 | 1 |
| 11370 | Asthma confirmed | 0 | 1 |
| 100397 | Asthma control questionnaire | 0 | 1 |
| 16785 | Asthma control step 1 | 0 | 1 |
| 16667 | Asthma control step 2 | 0 | 1 |
| 18224 | Asthma control step 3 | 0 | 1 |
| 20886 | Asthma control step 4 | 0 | 1 |
| 20860 | Asthma control step 5 | 0 | 1 |
| 98185 | Asthma control test | 0 | 1 |
| 42824 | Asthma daytime symptoms | 0 | 1 |
| 7416 | Asthma disturbing sleep | 0 | 1 |
| 13175 | Asthma disturbs sleep frequently | 0 | 1 |
| 38146 | Asthma disturbs sleep weekly | 0 | 1 |
| 13176 | Asthma follow-up | 0 | 1 |
| 7191 | Asthma limiting activities | 0 | 1 |
| 1002713 | Asthma limits activities 1 to 2 times per month | 0 | 1 |
| 102888 | Asthma limits activities 1 to 2 times per week | 0 | 1 |
| 103998 | Asthma limits activities most days | 0 | 1 |
| 38145 | Asthma limits walking on the flat | 0 | 1 |
| 38144 | Asthma limits walking up hills or stairs | 0 | 1 |
| 7378 | Asthma management plan given | 0 | 1 |
| 10274 | Asthma medication review | 0 | 1 |
| 25707 | Asthma monitor 1st letter | 0 | 1 |
| 25706 | Asthma monitor 2nd letter | 0 | 1 |
| 25705 | Asthma monitor 3rd letter | 0 | 1 |
| 31135 | Asthma monitor phone invite | 0 | 1 |
| 37943 | Asthma monitor verbal invite | 0 | 1 |
| 8355 | Asthma monitored | 0 | 1 |
| 81 | Asthma monitoring | 0 | 1 |
| 16655 | Asthma monitoring admin. | 0 | 1 |
| 30382 | Asthma monitoring admin.NOS | 0 | 1 |
| 30458 | Asthma monitoring by doctor | 0 | 1 |
| 19167 | Asthma monitoring by nurse | 0 | 1 |
| 19539 | Asthma monitoring check done | 0 | 1 |
| 18141 | Asthma monitoring due | 0 | 1 |
| 26501 | Asthma never causes daytime symptoms | 0 | 1 |
| 103612 | Asthma never causes night symptoms | 0 | 1 |
| 38143 | Asthma never disturbs sleep | 0 | 1 |
| 26504 | Asthma never restricts exercise | 0 | 1 |
| 31167 | Asthma night-time symptoms | 0 | 1 |
| 16070 | Asthma NOS | 0 | 1 |
| 13173 | Asthma not disturbing sleep | 0 | 1 |
| 13174 | Asthma not limiting activities | 0 | 1 |
| 7229 | Asthma prophylactic medication used | 0 | 1 |
| 10996 | Asthma resolved | 0 | 1 |
| 11839 | Asthma resolved | 0 | 1 |
| 25181 | Asthma restricts exercise | 0 | 1 |
| 102170 | Asthma review using Roy Colleg of Physicians thr | 0 | 1 |
| 5609 | Asthma screening | 0 | 1 |
| 105674 | Asthma self-management plan agreed | 0 | 1 |
| 26506 | Asthma severely restricts exercise | 0 | 1 |
| 13064 | Asthma severity | 0 | 1 |
| 26861 | Asthma sometimes restricts exercise | 0 | 1 |
| 19520 | Asthma treatment compliance satisfactory | 0 | 1 |
| 19519 | Asthma treatment compliance unsatisfactory | 0 | 1 |
| 11022 | Asthma trigger | 0 | 1 |
| 103944 | Asthma trigger - airborne dust | 0 | 1 |
| 103321 | Asthma trigger - animals | 0 | 1 |
| 103813 | Asthma trigger - cold air | 0 | 1 |
| 103945 | Asthma trigger - damp | 0 | 1 |
| 103952 | Asthma trigger - emotion | 0 | 1 |
| 102871 | Asthma trigger - exercise | 0 | 1 |
| 102341 | Asthma trigger - pollen | 0 | 1 |
| 102449 | Asthma trigger - respiratory infection | 0 | 1 |
| 102301 | Asthma trigger - seasonal | 0 | 1 |
| 103955 | Asthma trigger - tobacco smoke | 0 | 1 |
| 102952 | Asthma trigger - warm air | 0 | 1 |
| 4442 | Asthma unspecified | 0 | 1 |
| 96931 | At risk of chronic obstructive pulmonary diseas | 0 | 1 |
| 10403 | At risk of chronic obstructive pulmonary disease | 0 | 1 |
| 46529 | Attends asthma monitoring | 0 | 1 |
| 12953 | Attends stop smoking monitor. | 0 | 1 |
| 726 | Atypical chest pain | 0 | 1 |
| 102481 | Auscultation of lower respiratory tract | 0 | 1 |
| 57759 | Borg Breathlessness Score: 2 slight | 0 | 1 |
| 57193 | Borg Breathlessness Score: 3 moderate | 0 | 1 |
| 70061 | Borg Breathlessness Score: 7 very severe | 0 | 1 |
| 31143 | Breathless - at rest | 0 | 1 |
| 7683 | Breathless - lying flat | 0 | 1 |
| 7932 | Breathless - mild exertion | 0 | 1 |
| 6326 | Breathless - moderate exertion | 0 | 1 |
| 24889 | Breathless - strenuous exertion | 0 | 1 |
| 1429 | Breathlessness | 0 | 1 |
| 5175 | Breathlessness symptom | 0 | 1 |
| 20958 | Brief examn. of resp. system | 0 | 1 |
| 1555 | Bronchial asthma | 0 | 1 |
| 1025 | Bronchial cough | 0 | 1 |
| 2195 | Bronchiectasis | 0 | 1 |
| 32679 | Bronchiectasis NOS | 0 | 1 |
| 148 | Bronchitis unspecified | 0 | 1 |
| 104384 | Bronchodilator used infrequently | 0 | 1 |
| 3378 | Bronchodilators used a maximum of once daily | 0 | 1 |
| 26502 | Bronchodilators used more than once daily | 0 | 1 |
| 1273 | C/O - cough | 0 | 1 |
| 9552 | Change in asthma management plan | 0 | 1 |
| 95617 | Chest clear | 0 | 1 |
| 24704 | Chest wall pain | 0 | 1 |
| 24321 | Chest wall tenderness | 0 | 1 |
| 190 | Chest X-ray normal | 0 | 1 |
| 292 | Chesty cough | 0 | 1 |
| 46498 | Chr.obst. pulm. dis. screen | 0 | 1 |
| 106805 | Chronic asthma with fixed airflow obstruction | 0 | 1 |
| 5798 | Chronic asthmatic bronchitis | 0 | 1 |
| 3243 | Chronic bronchitis | 0 | 1 |
| 15626 | Chronic catarrhal bronchitis | 0 | 1 |
| 805 | Chronic catarrhal rhinitis | 0 | 1 |
| 1612 | Chronic cough | 0 | 1 |
| 4433 | Chronic maxillary sinusitis | 0 | 1 |
| 103678 | Chronic obstructiv pulmonary disease medication | 0 | 1 |
| 998 | Chronic obstructive airways disease | 0 | 1 |
| 5710 | Chronic obstructive airways disease NOS | 0 | 1 |
| 1001 | Chronic obstructive pulmonary disease | 0 | 1 |
| 102685 | Chronic obstructive pulmonary disease 3 monthly | 0 | 1 |
| 103007 | Chronic obstructive pulmonary disease 6 monthly | 0 | 1 |
| 11287 | Chronic obstructive pulmonary disease annual rev | 0 | 1 |
| 100237 | Chronic obstructive pulmonary disease assessment | 0 | 1 |
| 105457 | Chronic obstructive pulmonary disease care pathw | 0 | 1 |
| 45777 | Chronic obstructive pulmonary disease clini mana | 0 | 1 |
| 45770 | Chronic obstructive pulmonary disease disturbs s | 0 | 1 |
| 45771 | Chronic obstructive pulmonary disease does not d | 0 | 1 |
| 19428 | Chronic obstructive pulmonary disease excluded b | 0 | 1 |
| 18621 | Chronic obstructive pulmonary disease follow-up | 0 | 1 |
| 19721 | Chronic obstructive pulmonary disease leaflet gi | 0 | 1 |
| 38074 | Chronic obstructive pulmonary disease monitor ph | 0 | 1 |
| 9520 | Chronic obstructive pulmonary disease monitoring | 0 | 1 |
| 37247 | Chronic obstructive pulmonary disease NOS | 0 | 1 |
| 106945 | Chronic obstructive pulmonary disease rescue pack | 0 | 1 |
| 15782 | Chronic pulmonary heart disease NOS | 0 | 1 |
| 3244 | Chronic resp. dis. monitoring | 0 | 1 |
| 774 | Chronic rhinitis | 0 | 1 |
| 14645 | Chronic rhinitis NOS | 0 | 1 |
| 10546 | Chronic rhinosinusitis | 0 | 1 |
| 2257 | Chronic sinusitis | 0 | 1 |
| 5437 | Chronic sinusitis NOS | 0 | 1 |
| 94946 | Chronic type 1 respiratory failure | 0 | 1 |
| 94486 | Chronic type 2 respiratory failure | 0 | 1 |
| 42573 | Clear sputum | 0 | 1 |
| 100877 | Clinical chronic obstructive pulmonary disease q | 0 | 1 |
| 101385 | Consent given for follow-up by smoking cessation | 0 | 1 |
| 97800 | COPD - enhanced services administration | 0 | 1 |
| 18476 | COPD follow-up | 0 | 1 |
| 99948 | COPD patient unsuitable for pulmonary rehab - en | 0 | 1 |
| 104117 | COPD self-management plan agreed | 0 | 1 |
| 18501 | COPD self-management plan given | 0 | 1 |
| 104169 | COPD self-management plan review | 0 | 1 |
| 98283 | COPD structured smoking assessment declined - en | 0 | 1 |
| 92 | Cough | 0 | 1 |
| 7707 | Cough symptom NOS | 0 | 1 |
| 6134 | CXR - screening | 0 | 1 |
| 101851 | Declined consent for follow-up by smoking cessat | 0 | 1 |
| 106611 | Did not complete pulmonary rehabilitation progra | 0 | 1 |
| 2931 | Difficulty breathing | 0 | 1 |
| 32516 | Discharge by chest physician | 0 | 1 |
| 43401 | Discharge by respiratory physician | 0 | 1 |
| 30308 | DNA - Did not attend asthma clinic | 0 | 1 |
| 11527 | DNA - Did not attend smoking cessation clinic | 0 | 1 |
| 4931 | Dry cough | 0 | 1 |
| 5896 | Dyspnoea - symptom | 0 | 1 |
| 24479 | Emergency asthma admission since last appointmen | 0 | 1 |
| 19903 | Emergency COPD admission since last appointment | 0 | 1 |
| 794 | Emphysema | 0 | 1 |
| 33450 | Emphysema NOS | 0 | 1 |
| 14798 | Emphysematous bronchitis | 0 | 1 |
| 104608 | End stage chronic obstructive airways disease | 0 | 1 |
| 17828 | Examn. of respiratory system | 0 | 1 |
| 11695 | Excepted from asthma quality indicators: Informe | 0 | 1 |
| 11673 | Excepted from asthma quality indicators: Patient | 0 | 1 |
| 11266 | Excepted from COPD quality indicators: Informed | 0 | 1 |
| 11038 | Excepted from COPD quality indicators: Patient u | 0 | 1 |
| 30644 | Excepted from smoking quality indicators: Patient unsuitable | 0 | 1 |
| 18692 | Exception reporting: asthma quality indicators | 0 | 1 |
| 18717 | Exception reporting: COPD quality indicators | 0 | 1 |
| 5867 | Exercise induced asthma | 0 | 1 |
| 4606 | Exercise induced asthma | 0 | 1 |
| 12956 | Ex-heavy smoker (20-39/day) | 0 | 1 |
| 14454 | Expected FEV1 | 0 | 1 |
| 100963 | Ex-smoker annual review | 0 | 1 |
| 101338 | Failed attempt to stop smoking | 0 | 1 |
| 8512 | FEV1/FVC percent | 0 | 1 |
| 14456 | FEV1/FVC ratio | 0 | 1 |
| 88887 | FEV1/VC percent | 0 | 1 |
| 12104 | Flu vaccination administration | 0 | 1 |
| 22978 | Follow-up resp. assessment | 0 | 1 |
| 26186 | Forced expiratory flow rate between 25+75% of vi | 0 | 1 |
| 14453 | Forced expiratory volume - FEV | 0 | 1 |
| 10320 | Forced expired volume in 1 second | 0 | 1 |
| 10873 | Forced vital capacity - FVC | 0 | 1 |
| 97571 | Forced vital capacity before bronchodilation | 0 | 1 |
| 30758 | Good compliance with inhaler | 0 | 1 |
| 104265 | GP OOH service notified of COPD care plan | 0 | 1 |
| 719 | H/O: asthma | 0 | 1 |
| 4519 | H/O: bronchitis | 0 | 1 |
| 16342 | H/O: chr.obstr. airway disease | 0 | 1 |
| 7177 | H/O: respiratory disease | 0 | 1 |
| 104481 | Has chronic obstructive pulmonary disease care p | 0 | 1 |
| 2111 | Health ed. - smoking | 0 | 1 |
| 26496 | Health education - asthma | 0 | 1 |
| 100107 | Health education - asthma self management | 0 | 1 |
| 42313 | Health education - chronic obstructive pulmonary | 0 | 1 |
| 3568 | Heavy smoker - 20-39 cigs/day | 0 | 1 |
| 99762 | History of acute lower respiratory tract infecti | 0 | 1 |
| 103494 | History of chronic obstructive pulmonary disease | 0 | 1 |
| 26509 | Home nebuliser | 0 | 1 |
| 21855 | Home oxygen supply | 0 | 1 |
| 26435 | Home oxygen supply - concentrator | 0 | 1 |
| 39149 | Home oxygen support | 0 | 1 |
| 101116 | HOOF (home oxygen order form) completed | 0 | 1 |
| 103180 | Hypoxic challenge test | 0 | 1 |
| 45993 | Incentive spirometry | 0 | 1 |
| 31235 | Influenza imm.advised at home | 0 | 1 |
| 10584 | Influenza imm.advised in surg. | 0 | 1 |
| 4517 | Influenza immunization advised | 0 | 1 |
| 10821 | Influenza vacc consent given | 0 | 1 |
| 9039 | Influenza vacc. administratn. | 0 | 1 |
| 35655 | Influenza vacc.administrat.NOS | 0 | 1 |
| 6 | Influenza vaccination | 0 | 1 |
| 10674 | Influenza vaccination declined | 0 | 1 |
| 107024 | Influenza vaccination first telephone invitation | 0 | 1 |
| 106764 | Influenza vaccination first verbal invitation | 0 | 1 |
| 97941 | Influenza vaccination given by other healthcare | 0 | 1 |
| 11851 | Inhalation therapy | 0 | 1 |
| 13063 | Inhaled steroids use | 0 | 1 |
| 93393 | Inhaler device in use | 0 | 1 |
| 8484 | Inhaler technique - good | 0 | 1 |
| 13118 | Inhaler technique - moderate | 0 | 1 |
| 11758 | Inhaler technique - poor | 0 | 1 |
| 104450 | Inhaler technique not checked | 0 | 1 |
| 10711 | Inhaler technique observed | 0 | 1 |
| 6254 | Inhaler technique shown | 0 | 1 |
| 13172 | Initial resp. assessment | 0 | 1 |
| 8317 | Interstitial lung disease NEC | 0 | 1 |
| 45073 | Intrinsic asthma NOS | 0 | 1 |
| 101042 | Issue of chronic obstructive pulmonary disease rescue pack | 0 | 1 |
| 12987 | Late-onset asthma | 0 | 1 |
| 18926 | Lifestyle advice regarding smoking | 0 | 1 |
| 21432 | Loan of nebulizer | 0 | 1 |
| 57837 | Long term oxygen assessment | 0 | 1 |
| 21973 | Lung disease due to external agents | 0 | 1 |
| 1813 | Lung disease NOS | 0 | 1 |
| 23236 | Lung function signific. obstr. | 0 | 1 |
| 39617 | Lung function testing done | 0 | 1 |
| 852 | Lung function tests | 0 | 1 |
| 16567 | Lung volume test | 0 | 1 |
| 1665 | Medication review | 0 | 1 |
| 8034 | Medication review | 0 | 1 |
| 11955 | Medication review done | 0 | 1 |
| 11843 | Medication review with patient | 0 | 1 |
| 3018 | Mild asthma | 0 | 1 |
| 10863 | Mild chronic obstructive pulmonary disease | 0 | 1 |
| 25796 | Mixed asthma | 0 | 1 |
| 13065 | Moderate asthma | 0 | 1 |
| 10802 | Moderate chronic obstructive pulmonary disease | 0 | 1 |
| 42626 | Monitoring of all medication checked | 0 | 1 |
| 19432 | MRC Breathlessness Scale: grade 1 | 0 | 1 |
| 19427 | MRC Breathlessness Scale: grade 2 | 0 | 1 |
| 19426 | MRC Breathlessness Scale: grade 3 | 0 | 1 |
| 19430 | MRC Breathlessness Scale: grade 4 | 0 | 1 |
| 19429 | MRC Breathlessness Scale: grade 5 | 0 | 1 |
| 46036 | Multiple COPD emergency hospital admissions | 0 | 1 |
| 21610 | Nebulisation since last appointment | 0 | 1 |
| 11606 | Nebuliser at home | 0 | 1 |
| 6675 | Nebuliser therapy | 0 | 1 |
| 26439 | Nebuliser therapy | 0 | 1 |
| 57483 | Nebuliser ventilation | 0 | 1 |
| 37845 | Negative reversibility test to corticosteroid | 0 | 1 |
| 56691 | Negative reversibility test to ipratropium bromi | 0 | 1 |
| 10801 | Negative reversibility test to salbutamol | 0 | 1 |
| 34126 | Negotiated date for cessation of smoking | 0 | 1 |
| 9833 | Nicotine replacement therapy | 0 | 1 |
| 25106 | Nicotine replacement therapy provided free | 0 | 1 |
| 85975 | Nicotine replacement therapy using nicotine gum | 0 | 1 |
| 85247 | Nicotine replacement therapy using nicotine inha | 0 | 1 |
| 89464 | Nicotine replacement therapy using nicotine loze | 0 | 1 |
| 81440 | Nicotine replacement therapy using nicotine patc | 0 | 1 |
| 19346 | No breathlessness | 0 | 1 |
| 59840 | No nebulisation since last appointment | 0 | 1 |
| 28982 | No peak flow meter at home | 0 | 1 |
| 30552 | No respiratory symptoms | 0 | 1 |
| 4836 | Nocturnal cough / wheeze | 0 | 1 |
| 98177 | Non-smoker annual review - enhanced services adm | 0 | 1 |
| 26508 | Not using inhaled steroids | 0 | 1 |
| 9018 | Number of asthma exacerbations in past year | 0 | 1 |
| 28743 | Number of COPD exacerbations in past year | 0 | 1 |
| 40566 | Number of times bronchodilator used in 24 hours | 0 | 1 |
| 2909 | O/E - breath sounds | 0 | 1 |
| 21407 | O/E - breath sounds normal | 0 | 1 |
| 9496 | O/E - breathing description | 0 | 1 |
| 11614 | O/E - bronchial breathing | 0 | 1 |
| 1692 | O/E - bronchospasm | 0 | 1 |
| 3318 | O/E - chest examination normal | 0 | 1 |
| 20046 | O/E - chest expansion normal | 0 | 1 |
| 8582 | O/E - chest findings | 0 | 1 |
| 7000 | O/E - dyspnoea | 0 | 1 |
| 5861 | O/E - expiratory wheeze | 0 | 1 |
| 17992 | O/E - normal respiration | 0 | 1 |
| 24343 | O/E - resp. examination NOS | 0 | 1 |
| 7534 | O/E - respiratory distress | 0 | 1 |
| 14465 | O/E - respiratory rate | 0 | 1 |
| 27819 | Obstructive chronic bronchitis | 0 | 1 |
| 22752 | Occupational asthma | 0 | 1 |
| 28297 | Oral steroids used since last appointment | 0 | 1 |
| 40788 | Other emphysema | 0 | 1 |
| 18487 | Other lung anomalies | 0 | 1 |
| 7202 | Other mycobacterial diseases | 0 | 1 |
| 12403 | Other respiratory disease monitoring | 0 | 1 |
| 38987 | Other specified diseases of respiratory system | 0 | 1 |
| 42929 | Other specified diseases of respiratory system | 0 | 1 |
| 96603 | Other specified other respiratory support | 0 | 1 |
| 91708 | Other specified smoking cessation therapy | 0 | 1 |
| 8417 | Oxygen at home | 0 | 1 |
| 6877 | Oxygen therapy | 0 | 1 |
| 5211 | Oxygen therapy | 0 | 1 |
| 55888 | Oxygen therapy | 0 | 1 |
| 32961 | Oxygen therapy | 0 | 1 |
| 100132 | Oxygen therapy follow up assessment | 0 | 1 |
| 100154 | Oxygen therapy initial assessment | 0 | 1 |
| 106852 | Oxygen therapy percentage | 0 | 1 |
| 9598 | Oxygenator therapy | 0 | 1 |
| 99793 | Patient has a written asthma personal action pla | 0 | 1 |
| 84 | Peak exp. flow rate: PEFR/PFR | 0 | 1 |
| 61417 | Peak expiratory flow rate - compliance moderate | 0 | 1 |
| 25792 | Peak expiratory flow rate - technique good | 0 | 1 |
| 47053 | Peak expiratory flow rate - technique moderate | 0 | 1 |
| 46658 | Peak expiratory flow rate - technique poor | 0 | 1 |
| 11668 | Peak expiratory flow rate monitoring | 0 | 1 |
| 11671 | Peak expiratory flow rate monitoring using diary | 0 | 1 |
| 12697 | Peak flow meter at home | 0 | 1 |
| 11772 | Peak flow rate | 0 | 1 |
| 11832 | Peak flow rate abnormal | 0 | 1 |
| 14232 | Peak flow rate after bronchodilation | 0 | 1 |
| 14231 | Peak flow rate before bronchodilation | 0 | 1 |
| 14227 | Peak flow rate normal | 0 | 1 |
| 100275 | Peak inspiratory flow rate | 0 | 1 |
| 6723 | PEFR - peak exp. flow rate | 0 | 1 |
| 28949 | PEFR monitoring using diary | 0 | 1 |
| 6091 | Percent predicted FEV1 | 0 | 1 |
| 1468 | Perennial rhinitis | 0 | 1 |
| 3628 | Persistent cough | 0 | 1 |
| 1835 | PFR - peak flow rate | 0 | 1 |
| 102492 | Pleural plaque | 0 | 1 |
| 5005 | Pleural plaque disease due to asbestosis | 0 | 1 |
| 10877 | Pneumococcal immunisation advised in surgery | 0 | 1 |
| 11363 | Pneumococcal vaccination | 0 | 1 |
| 30411 | Pneumococcal vaccination administration | 0 | 1 |
| 10686 | Pneumococcal vaccination declined | 0 | 1 |
| 36826 | Pneumococcal vaccination given | 0 | 1 |
| 10086 | Pneumonia and influenza | 0 | 1 |
| 6094 | Pneumonia or influenza NOS | 0 | 1 |
| 7731 | Pollen asthma | 0 | 1 |
| 102522 | Post bronchodilator spirometry | 0 | 1 |
| 30509 | Post operative chest infection | 0 | 1 |
| 101764 | Practice based smoking cessation programme start | 0 | 1 |
| 7706 | Productive cough -clear sputum | 0 | 1 |
| 7773 | Productive cough -green sputum | 0 | 1 |
| 1234 | Productive cough NOS | 0 | 1 |
| 7708 | Productive cough-yellow sputum | 0 | 1 |
| 103472 | Pulmonary fibrosis | 0 | 1 |
| 30647 | Pulmonary rehabilitation | 0 | 1 |
| 43477 | Pulmonary rehabilitation class | 0 | 1 |
| 94952 | Pulmonary rehabilitation declined | 0 | 1 |
| 46844 | Pulmonary rehabilitation programme commenced | 0 | 1 |
| 10845 | Pulmonary rehabilitation programme completed | 0 | 1 |
| 4899 | Recurrent chest infection | 0 | 1 |
| 17173 | Recurrent sinusitis | 0 | 1 |
| 86990 | Reduction of lung volume | 0 | 1 |
| 98284 | Refer COPD structured smoking assessment - enhan | 0 | 1 |
| 54015 | Referral for long-term oxygen therapy assessmt n | 0 | 1 |
| 102361 | Referral for smoking cessation service offered | 0 | 1 |
| 13683 | Referral for spirometry | 0 | 1 |
| 10181 | Referral to chest physician | 0 | 1 |
| 103758 | Referral to COPD community nursing team | 0 | 1 |
| 102622 | Referral to home oxygen service | 0 | 1 |
| 98154 | Referral to NHS stop smoking service | 0 | 1 |
| 40735 | Referral to pulmonary rehabilitation | 0 | 1 |
| 103488 | Referral to respiratory clinic declined | 0 | 1 |
| 19261 | Referral to respiratory nurse specialist | 0 | 1 |
| 10874 | Referral to respiratory physician | 0 | 1 |
| 95895 | Referral to respiratory physician | 0 | 1 |
| 25703 | Referral to respiratory rapid response team | 0 | 1 |
| 18573 | Referral to smoking cessation advisor | 0 | 1 |
| 106359 | Referral to smoking cessation service | 0 | 1 |
| 106391 | Referral to smoking cessation service declined | 0 | 1 |
| 10742 | Referral to stop-smoking clinic | 0 | 1 |
| 46088 | Referred for assessment of need for oxygen thera | 0 | 1 |
| 103400 | Referred for COPD structured smoking assessment | 0 | 1 |
| 8264 | Referred to acute chest pain clinic | 0 | 1 |
| 11387 | Refuses asthma monitoring | 0 | 1 |
| 40418 | Refuses stop smoking monitor | 0 | 1 |
| 22404 | Rep.presc. monitoring NOS | 0 | 1 |
| 16575 | Rep.presc. treatment changed | 0 | 1 |
| 29293 | Rep.presc.treatment started | 0 | 1 |
| 5982 | Rep.presc.treatment stopped | 0 | 1 |
| 13062 | Resp. disease monitoring NOS | 0 | 1 |
| 47935 | Resp. disease screen NOS | 0 | 1 |
| 42658 | Resp. drug side effects | 0 | 1 |
| 860 | Resp. system examined - NAD | 0 | 1 |
| 29999 | Resp. treatment changed | 0 | 1 |
| 22052 | Resp.dis.treatment started | 0 | 1 |
| 6391 | Respiration monitoring | 0 | 1 |
| 1608 | Respiratory disease monitoring | 0 | 1 |
| 8798 | Respiratory disease screen | 0 | 1 |
| 51476 | Respiratory education | 0 | 1 |
| 7074 | Respiratory infection NOS | 0 | 1 |
| 53173 | Respiratory medication | 0 | 1 |
| 9921 | Respiratory monitoring | 0 | 1 |
| 22649 | Respiratory monitoring NOS | 0 | 1 |
| 19750 | Respiratory physiotherapy | 0 | 1 |
| 23663 | Respiratory symptom NOS | 0 | 1 |
| 5818 | Respiratory symptoms | 0 | 1 |
| 25840 | Respiratory symptoms NOS | 0 | 1 |
| 5733 | Respiratory system diseases | 0 | 1 |
| 69289 | Respiratory tests | 0 | 1 |
| 38077 | Reversibility trial by anticholinergic | 0 | 1 |
| 28844 | Reversibility trial by bronchodilator | 0 | 1 |
| 22041 | Reversibility trial by steroids | 0 | 1 |
| 107336 | Review of inhaler technique using inhaler checki | 0 | 1 |
| 12945 | Rolls own cigarettes | 0 | 1 |
| 93736 | Royal College of Physicians asthma assessment | 0 | 1 |
| 103631 | Royal College Physician asthma assessment 3 ques | 0 | 1 |
| 10634 | Salbutamol reversibility | 0 | 1 |
| 10839 | Seen by chest physician | 0 | 1 |
| 103716 | Seen by respiratory nurse specialist | 0 | 1 |
| 8269 | Seen by respiratory physician | 0 | 1 |
| 18885 | Seen by respiratory physician | 0 | 1 |
| 11356 | Seen by smoking cessation advisor | 0 | 1 |
| 37799 | Seen by thoracic physician | 0 | 1 |
| 22429 | Seen by thoracic surgeon | 0 | 1 |
| 5515 | Seen in asthma clinic | 0 | 1 |
| 3353 | Seen in chest clinic | 0 | 1 |
| 85504 | Seen in community respiratory clinic | 0 | 1 |
| 6968 | Seen in oral surgery clinic | 0 | 1 |
| 94193 | Seen in respiratory clinic | 0 | 1 |
| 94700 | Seen in thoracic medicine clinic | 0 | 1 |
| 27776 | Seen in thoracic surgery clinic | 0 | 1 |
| 7590 | Seen in thoracic surgery clinic | 0 | 1 |
| 9876 | Severe chronic obstructive pulmonary disease | 0 | 1 |
| 5349 | Shortness of breath symptom | 0 | 1 |
| 1823 | Smoker | 0 | 1 |
| 16717 | Smokers' cough | 0 | 1 |
| 96992 | Smoking cessation - enhanced services administra | 0 | 1 |
| 7622 | Smoking cessation advice | 0 | 1 |
| 100099 | Smoking cessation advice declined | 0 | 1 |
| 41042 | Smoking cessation advice provided by community p | 0 | 1 |
| 94958 | Smoking cessation drug therapy | 0 | 1 |
| 104185 | Smoking cessation drug therapy declined | 0 | 1 |
| 10211 | Smoking cessation milestones | 0 | 1 |
| 104230 | Smoking cessation programme declined | 0 | 1 |
| 38112 | Smoking cessation programme start date | 0 | 1 |
| 74907 | Smoking cessation therapy | 0 | 1 |
| 90522 | Smoking cessation therapy NOS | 0 | 1 |
| 10898 | Smoking free weeks | 0 | 1 |
| 12966 | Smoking reduced | 0 | 1 |
| 41979 | Smoking restarted | 0 | 1 |
| 12952 | Smoking started | 0 | 1 |
| 34127 | Smoking status at 4 weeks | 0 | 1 |
| 34374 | Smoking status between 4 and 52 weeks | 0 | 1 |
| 25944 | Spacer device in use | 0 | 1 |
| 103593 | Spirometric lung age | 0 | 1 |
| 6118 | Spirometry | 0 | 1 |
| 29015 | Spirometry | 0 | 1 |
| 101053 | Spirometry contraindicated | 0 | 1 |
| 26241 | Spirometry indicated | 0 | 1 |
| 12827 | Spirometry not indicated | 0 | 1 |
| 10336 | Spirometry reversibility | 0 | 1 |
| 10420 | Spirometry reversibility negative | 0 | 1 |
| 10492 | Spirometry reversibility positive | 0 | 1 |
| 30674 | Spirometry reversibility testing contraindicated | 0 | 1 |
| 89207 | Spirometry reversibility testing not indicated | 0 | 1 |
| 10337 | Spirometry screening | 0 | 1 |
| 26349 | Spirometry test declined | 0 | 1 |
| 9807 | Sputum - symptom | 0 | 1 |
| 14273 | Sputum appearance | 0 | 1 |
| 18964 | Sputum clearance | 0 | 1 |
| 14271 | Sputum culture | 0 | 1 |
| 14518 | Sputum cytology | 0 | 1 |
| 8287 | Sputum sample obtained | 0 | 1 |
| 21333 | Standard chest X-ray requested | 0 | 1 |
| 9663 | Step up change in asthma management plan | 0 | 1 |
| 1577 | Steroid therapy | 0 | 1 |
| 100976 | Steroid treatment card issued | 0 | 1 |
| 32083 | Stop smoking clinic admin. | 0 | 1 |
| 98245 | Stop smoking face to face follow-up | 0 | 1 |
| 42722 | Stop smoking monitor 1st lettr | 0 | 1 |
| 21637 | Stop smoking monitor admin.NOS | 0 | 1 |
| 58597 | Stop smoking monitor phone inv | 0 | 1 |
| 19485 | Stop smoking monitor.chck done | 0 | 1 |
| 7130 | Stop smoking monitoring admin. | 0 | 1 |
| 103507 | Stop smoking service opportunity signposted | 0 | 1 |
| 10318 | Suspected asthma | 0 | 1 |
| 19434 | Suspected chronic obstructive pulmonary disease | 0 | 1 |
| 30423 | Thinking about stopping smoking | 0 | 1 |
| 32601 | Under care of chest physician | 0 | 1 |
| 58170 | Under care of community respiratory team | 0 | 1 |
| 32622 | Under care of respiratory physician | 0 | 1 |
| 107318 | Under care of respiratory physician | 0 | 1 |
| 45586 | Using inhaled steroids - high dose | 0 | 1 |
| 107641 | Using inhaled steroids - low dose | 0 | 1 |
| 26505 | Using inhaled steroids - normal dose | 0 | 1 |
| 93568 | Very severe chronic obstructive pulmonary diseas | 0 | 1 |
| 6421 | Viral upper respiratory tract infection NOS | 0 | 1 |
| 19828 | Vital capacity test | 0 | 1 |
| 2891 | Wheezing symptom | 0 | 1 |
| 152 | Wheezy bronchitis | 0 | 1 |
